# Cep63 and Cep152 Cooperate to Ensure Centriole Duplication

**DOI:** 10.1371/journal.pone.0069986

**Published:** 2013-07-30

**Authors:** Nicola J. Brown, Marko Marjanović, Jens Lüders, Travis H. Stracker, Vincenzo Costanzo

**Affiliations:** 1 Cancer Research UK London Research Institute, Clare Hall Laboratories, South Mimms, United Kingdom; 2 Institute for Research in Biomedicine (IRB Barcelona), Barcelona, Spain; 3 Division of Molecular Medicine, Ruđer Bošković Institute, Zagreb, Croatia; Virginia Tech, United States of America

## Abstract

Centrosomes consist of two centrioles embedded in pericentriolar material and function as the main microtubule organising centres in dividing animal cells. They ensure proper formation and orientation of the mitotic spindle and are therefore essential for the maintenance of genome stability. Centrosome function is crucial during embryonic development, highlighted by the discovery of mutations in genes encoding centrosome or spindle pole proteins that cause autosomal recessive primary microcephaly, including Cep63 and Cep152. In this study we show that Cep63 functions to ensure that centriole duplication occurs reliably in dividing mammalian cells. We show that the interaction between Cep63 and Cep152 can occur independently of centrosome localisation and that the two proteins are dependent on one another for centrosomal localisation. Further, both mouse and human Cep63 and Cep152 cooperate to ensure efficient centriole duplication by promoting the accumulation of essential centriole duplication factors upstream of SAS-6 recruitment and procentriole formation. These observations describe the requirement for Cep63 in maintaining centriole number in dividing mammalian cells and further establish the order of events in centriole formation.

## Introduction

The centrosome is the primary microtubule organising centre in dividing cells and is composed of 2 centrioles that are embedded in pericentriolar material (PCM). Centrioles are cylindrical structures composed of triplet and doublet microtubules, arranged with a 9-fold radial symmetry [1]. In addition to their essential role in the formation of the centrosome, centrioles are also required for the assembly of cilia and flagella [1], [2]. Centriole number is under tight regulation in dividing cells. A newly born cell in the G1 phase of the cell cycle contains two disengaged centrioles, both competent to organise PCM and form a new procentriole at, and perpendicular to, the proximal end [3]. Procentriole formation occurs in a semiconservative fashion at the G1-S phase transition, and by G2 phase, cells contain two centrosomes, each with two centrioles that are tightly linked to each other, to ensure that during cell division each daughter cell receives a centrosome composed of two centrioles. The strict regulation of centriole number is crucial for the accurate and symmetrical formation of the mitotic spindle and precise, reproducible segregation of the genome during mitosis.

Key components of centriole biogenesis have been identified in genetic studies using *Caenorhabditis elegans* [4]. Work in flies and humans demonstrated that most of the key components in centriole biogenesis are functionally conserved despite poor conservation at the amino acid level [1]. *C. elegans* SPD-2 is the most upstream component of centriole biogenesis, required for the recruitment of the *C. elegans* polo-like kinase 4 (Plk4) functional equivalent, ZYG-1 [5]. The mammalian orthologue of SPD-2, Cep192, is required for centriole duplication but its role in the process has yet to be elucidated [6]. Plk4, the master regulator of centriole duplication, and Cep152 are essential for the early steps of procentriole formation [7]–[12]. The recruitment of the structural component SAS-6 dictates the 9-fold symmetry of the newly forming centriole [9], [11], [13]–[15]. In humans, procentriole formation and elongation further require the conserved components Cep135, STIL and CPAP, and a growing list of additional proteins [1], [16]–[19].

Aberrations in centriole structure or function are associated with severe human diseases including ciliopathies, cancer, and problems in embryonic development [2]. To date, mutations in nine genes encoding centrosome proteins have been identified in patients with primary microcephaly including some that are required for centriole duplication: STIL, CPAP, Cep152, Cep63 and Cep135 [20]–[24]. One hypothesis for the cause of primary microcephaly is that centrosome defects can lead to depletion of neuronal precursors due to defects in mitotic spindle positioning during stem cell divisions in the cortex of the brain during embryogenesis [16], [25], [26].

Cep63 was originally identified as a component of purified centrosomes by mass spectrometry and has since been demonstrated to play a role in centrosome-dependent assembly of bipolar mitotic spindles in *Xenopus laevis* egg extracts and in chicken DT40 cells [27], [28]. Recent studies in chicken DT40 cells have revealed a function for Cep63 in centriole duplication through its interaction with Cep152 [24]. The interaction between Cep63 and Cep152 is conserved in human cells, but whether Cep63 is required for the process of centriole duplication in mammalian cells has yet to be thoroughly investigated. Understanding the molecular functions of these centrosomal proteins in more detail will provide important insight regarding the aetiology of microcephalies and other human diseases resulting from defects in centriole and centrosome function.

In this study, we show that the N-terminal region of Cep63 is required for localisation of Cep63 and Cep152 to the centrosome, and that Cep63 and Cep152 interact independently of centrosome localisation, suggesting that Cep63 and Cep152 are recruited to the centrosome together. Additionally, we demonstrate that Cep63 is necessary for centriole duplication to occur efficiently in both mouse and human cells, and that Cep63 functions, together with Cep152, upstream of SAS-6 recruitment. Reduced levels of Cep63 and Cep152 at the centrosome led to a reduction in PCM size, impaired SAS-6 recruitment and inefficient centriole duplication. We conclude that Cep63 and Cep152 cooperate to ensure full recruitment of PCM components required for centriole duplication, thus playing an early role in centriole biogenesis, before the recruitment of the SAS-6 cartwheel.

## Materials and Methods

### Plasmids and siRNAs

IMAGE clone 5951988 (Cep63) and 40125733 (Cep152) were used as PCR templates for all plasmids generated, except pEGFP-Cep152, which was kindly provided by Ingrid Hoffmann (DKFZ, Germany). Cep63 was cloned into pMAL-c4x (NEB) for expression of MBP-Cep63 in *E. coli*. (GFP-Cep63 and GFP-Cep152-N terminal (nucleotides 1–2409) and –C terminal (nucleotides 2410–4965) cDNAs were cloned into the pIRESpuro3 vector (Clontech), modified with an N-terminal GFP-Flag-tag (a gift from Tohru Takaki, Clare Hall Laboratories). GFP-Cep63 W was generated by two-step site directed mutagenesis using pIRESpuro3GFP-Flag-Cep63 as the first template with primers 5′-AAATAGAGGAATTCCGTCAAAAGTCCCTGGACTGGGAGAAGCAAC-3′ and 5′-GTTGCTTCTCCCAGTCCAGGGACTTTTGACGGAATTCCTCTATTT-3′, then with the resulting plasmid as a template, the following primers were used: 5′- AAATAGAGGAATTCCGTCAAAAGAGCCTCGATTGGGAGAAGCAAC-3′ and 5′-GTTGCTTCTCCCAATCGAGGCTCTTTTGACGGAATTCCTCTATTT-3′. Cep63 full length and truncations 1–135 (nucleotides 1–408), 136–424 (409–1272), 425–541 (1273–1626), 1–424 (1–1272), and 136–541 (409–1626), and Cep152 were cloned into the Gateway system pDONR221 vector (Invitrogen), then into the Gateway destination vector pcDNA5FRT/TO (Invitrogen), modified with either an N-terminal YFP or Flag tag (gift from Zuzana Horejsi, Clare Hall Laboratories). siRNAs Cep63-1 and -2 and Cep152-1 and -2 were purchased from Dharmacon; (Control Medium GC content) and Cep63-3, from Invitrogen; and Cep192, from Eurofins. Cep63, Cep152, and Cep192 siRNA sequences are as follows:

63-1 5′-GAGUUACAUCAGCGAGAUA; 63-2 5′-CGUCAGAAAUCGCUGGACU; 63-3 5′-GGAGUCAGUUGGAUGUGACACAUAA; 152-1 5′-GCAUUGAGGUUGAGACUAA; 152-2 5′-GACCAGAGUCGUAGAGAAU; 192 5′-AGAGAUGAAAAUGUCUUCC as previously described [6].

### Cell Culture

HeLa Kyoto, U2OS and 293 FlpIn TREX cells (Invitrogen) were obtained from the London Research Institute Cell Services and were grown in DMEM (+4.5 g/l glucose+Glutamine+Pyruvate, GIBCO) with 10% heat inactivated foetal calf serum (FCS, PAA Laboratories GmbH). Plasmid DNA was transfected using Effectene (Qiagen) and siRNAs were transfected at 50 nM final concentration using Lipofectamine RNAiMAX (Invitrogen). RNAi was carried out for 4 days following a reverse transfection and one forwards transfection 24 to 48 hours later. U2OS GFP-Cep63 W, or GFP-empty vector, stable cell lines were generated by selection of resistant clones after incubation of transfected cells with 0.3 µg/ml Puromycin. HeLa Kyoto GFP-Cep63 stable cell lines were generated in the same way. 293 FlpIn Flag-Cep63 and YFP-Cep63, and 293 FlpIn TREX stable Flag-Cep63 (full length and truncations) cell lines were generated by selection of stable clones with 100 µg/ml Hygromycin B after transfection. Expression was induced by incubation with 2 µg/ml doxycycline in TREX cell lines. Primary MEFs were grown in DMEM (GIBCO) with 10% heat inactivated FCS (PAA Laboratories GmbH) supplemented with Penicillin (10 units/ml) and Streptomycin (100 µg/ml, GIBCO). All cell lines were grown at 37°C at 5% CO_2_. For centrosome reduplication experiments, cells were first transfected with siRNAs, then Aphidicolin (2 µg/ml) was added along with the second siRNA transfection after 24 hours then cells were collected after a further 72 hours.

### Cep63 Gene-trap Mice and MEF Generation

Mouse embryonic stem (ES) cells containing a gene-trap cassette between exons 1 and 2 of the Cep63 gene were purchased from EUCOMM (European Conditional Mouse Mutagenesis Program; Cep63 MGI 2158560; clone ID EUCE0251_H11) and injected into 3.5 day old mouse blastocysts derived from C57B6/j mice. Approximately 12–15 ES cells were injected into each blastocyst, and injected blasts were re-implanted back into the oviduct of 2.5 day pseudo-pregnant foster mice (CD1’s). Chimeras were scored by coat colour analysis, and the chimeras showing the highest contribution from the ES cells were mated with C57B6/j wild-type mice. Agouti offspring obtained from these test-matings were screened for the presence of the mutation. Subsequent pups were screened for the zygosity of the mutation using the common forward primer (5P2 5′-GTAGGACCAGGCCTTAGCGTTAG-3′) with a wild type specific (3P2 5′-TGAAACTTCAGCATATACAC-3′) or mutant specific (B32 5′-CAAGGCGATTAAGTTGGGTAACG-3′) reverse primer. For the preparation of MEF cultures, embryos were isolated at E14.5 and incubated overnight in trypsin following removal of tissue for genotyping. Embryos were disaggregated in media using a pipette and plated in DMEM with high glucose, 15% FBS and penicillin/streptomycin. Primary MEFs were spontaneously immortalised using a variation of the 3T3 protocol [29]. Briefly, 300,000 cells were plated on a 60 mm tissue culture plate and reseeded every 3 days. For centrosome reduplication assays, 3T3 cell lines were incubated with 2 µg/ml aphidicolin or 10 µM Cdk1 inhibitor (RO-3306, Santa Cruz) for 72 hours before fixation for IF. All animals were handled in strict accordance with the guidelines of the European Community (86/609/EEC) at the animal facilities in the Barcelona Science Park. The protocols were approved by the Animal Care and Use Committee of the Barcelona Science Park (IACUC; CEEA-PCB) in accordance with applicable legislation (Law 5/1995/GC; Order 214/1997/GC; Law 1201/2005/SG). All efforts were made to minimise suffering.

### Antibodies

**Figure 1:**
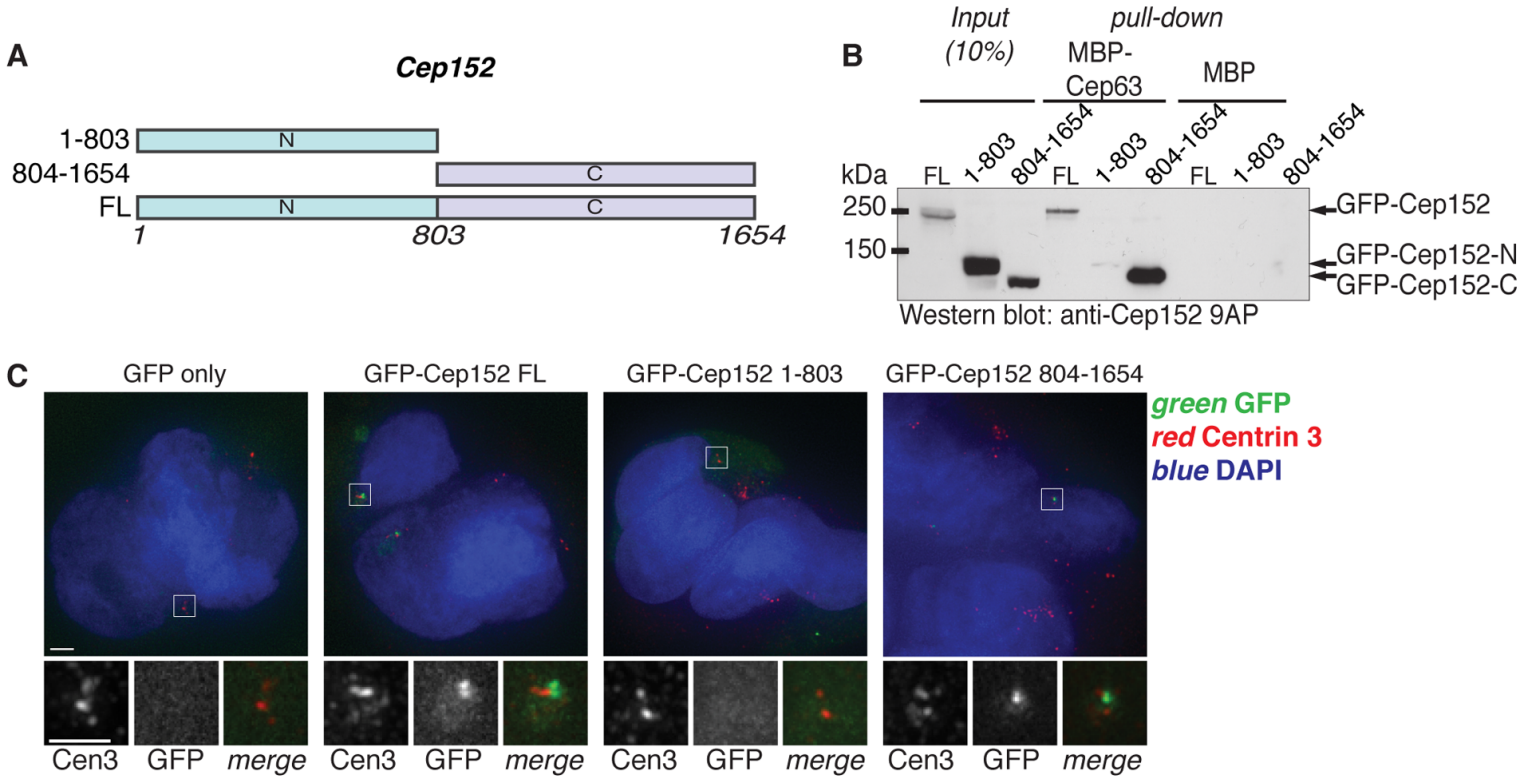
The C-terminal half of Cep152 is required for Cep63 binding and centrosomal localisation. (A) Diagram of Cep152 full length and truncation proteins used in the following experiments. Numbers indicate amino acid positions. (B) Cep63 interacts with the C-terminal half of Cep152. MBP or MBP-Cep63 pull down experiments were carried out after incubation in lysates of 293 HEK cells expressing GFP-Cep152 full length (FL), N-terminal half (1–803), or C-terminal half (804–1654), and Cep152 proteins were detected by Western blotting with anti-Cep152 antibodies (Cep152 9AP). Input shows 10% of cell lysate used for pull down experiments. (C) The C-terminal half of Cep152 is required for its centrosomal localisation. 293 HEK cells expressing the GFP-Cep152 proteins used in (B) were stained with DAPI (blue) and anti-Centrin 3 antibodies (red), GFP direct fluorescence is shown in green. Lower panels show magnification of one centrosome (boxed region). Scale bars 5 µm.

Commercial anti-α-tubulin mouse (Sigma-Aldrich), Centrin 2 rabbit (Santa Cruz), Centrin 3 mouse (Abcam), Cep63 rabbit (Millipore), Cep152 rabbit (no. 479, Bethyl), Cep192 (Bethyl), Flag M2 mouse (Sigma-Aldrich), γ-tubulin mouse (Sigma-Aldrich), Ninein rabbit (Abcam), Pericentrin rabbit (Covance), HsSAS-6 mouse (Santa Cruz) and PCNA mouse (PC10, Santa Cruz) antibodies were used for immunofluorescence and/or Western blotting. Anti-GFP rabbit serum was used for Western blotting and anti-Cyclin A mouse clone AT10 was used for immunofluorescence (both produced by Julian Gannon, Genome Stability Laboratory, Clare Hall). Antibodies were raised against a mixture of two Cep152 peptides (amino acids 18–31 and 1600–1617) in rabbit, which were used for Western blotting (Figure 1B) after affinity purification using both peptides. For Western blotting, secondary goat anti-mouse and goat anti-rabbit HRP-coupled antibodies were used (Dako). For immunofluorescence, secondary goat anti-mouse Alexa Fluor 488, 594, and 350, and goat anti-rabbit Alexa Fluor 488 and 594 antibodies were used.

### Western Blotting, Immuno-precipitations and Pull-down Assays

Cells were lysed in RIPA buffer (150 mM NaCl, 1% Igepal, 0.1% SDS, 0.5% sodium deoxycholate) plus Complete protease inhibitors (Roche). For Flag IPs, 1 mg cell lysate was incubated with 25 µl Flag M2 resin (Sigma-Aldrich) in 0.5 ml lysis buffer (50 mM Tris HCl, pH 7.4, 150 mM NaCl, 1 mMEDTA, 1% Triton X100) plus Complete protease inhibitors (Roche), for 2 hours at 4°C. Resin was washed with PBS; proteins were eluted from the resin by boiling in Laemmli buffer (Bio-Rad) then analysed by SDS-PAGE and Western blotting. For MBP or MBP-Cep63 pull down assays, 2 µg of MBP or MBP-Cep63 was incubated with 30 µl amylose resin in column buffer (20 mM Tris HCl pH7.5, 200 nM NaCl, 1 mM EDTA, 1 mM DTT) plus Complete protease inhibitors (Roche), per reaction, for 1 hour at 4°C. After washing in column buffer, amylose resin was incubated with 1 mg cell lysate from cells 293 expressing GFP-Cep152 full length, N-, or C-terminal domains, in lysis buffer for 2 hours at 4°C. Resin was washed with PBS, and proteins were eluted by boiling in Laemmli buffer (Bio-Rad). MBP and MBP-Cep63 were expressed in BL21 CodonPlus (DE3) RIL *E. coli* (Stratagene) at 16°C for 3 hours after induction with 0.3 mM IPTG (Isopropyl β-D-1-thiogalactopyranoside), and purified using amylose resin according the manufacturer’s protocol (NEB).

### Immunofluorescence and Fluorescence Microscopy

Cells were grown on glass coverslips; fixed in −20°C methanol for at least 10 minutes; rehydrated in PBS 0.01% Triton X-100 (TX); and permeabilised with PBS 0.2% TX. Coverslips were incubated with primary antibodies (as indicated) diluted in antibody blocking buffer overnight at 4°C; washed with PBS 0.01% TX; incubated with secondary Alexa Fluor antibodies (as indicated) in antibody blocking buffer for 1 hour at room temperature; washed with PBS 0.01% TX; incubated with PBS 0.01% TX containing 1 µg/ml DAPI (4,6- diamidino-2-phenylindole); then mounted with Vectashield (Vector Laboratories). For co-staining with two antibodies from the same species (Cep63 and Centrin 2; Cyclin A and HsSAS-6; PCNA and HsSAS-6) antibodies were incubated sequentially with extensive washing between the first secondary antibody and the second primary antibody. For Centrin-2 and Cep63 co-staining, the antibody incubations were done in the following order: anti-Centrin-2, goat anti-rabbit Alexa Fluor 594, anti-Cep63, goat anti-rabbit Alexa Fluor 488. For Cyclin A (or PCNA) and HsSAS-6 co-staining, the antibodies were used in the following order: anti-HsSAS-6, goat anti-mouse Alexa Fluor 594, anti-Cyclin A (or PCNA), goat anti-mouse Alexa Fluor 488. Analysis and image capture was carried out on a Delta Vision RT inverted fluorescence microscope with Softworx software, using an Olympus UPlanSApo 100×/1.4 oil objective and a COOLSNAPHQ/ICX285 CCD camera. Z-stacks were acquired at 0.2 µm intervals, then projected by maximum intensity to a flat image. Deconvolution was performed using Softworx with the “enhanced ratio (aggressive)” setting and noise filtering set to medium for 10 cycles. Fluorescence measurements were carried out using Image J using a circular region of interest with the area kept constant, recording the Integrated Density from raw Softworx files (R3D).

## Results

### Cep63-Cep152 Interaction is Required for Centrosomal Localisation of Both Proteins

Cep63 and Cep152 interact and localise to the pericentriolar material, forming toroid shapes surrounding the wall of the mother centriole [7], [8], [10], [24], [30], [31] (see also Figure S1). Cep63 is required for localisation of Cep152 to the centrosome and, conversely, Cep152 is required for localisation of Cep63 to the centrosome [24], [30] (also Figure S2).

We used *Xenopus laevis* cell free egg extracts, which lack centrosomes, to address whether Cep63 and Cep152 require localisation to the centrosome for their interaction [32]. Using recombinant proteins incubated in egg extracts, we found that the *Xenopus laevis* Cep63 and Cep152 orthologues interact, indicating that the Cep63-Cep152 interaction can occur independently of centrosome localisation (XCep63 and XCep152, Figure S1C). Furthermore, pull down of ^35^S-XCep63 with MBP-XCep152 after incubation *in vitro*, without the addition of extract, indicated that the interaction is likely to be direct (Figure S1C, last two lanes).

**Figure 2:**
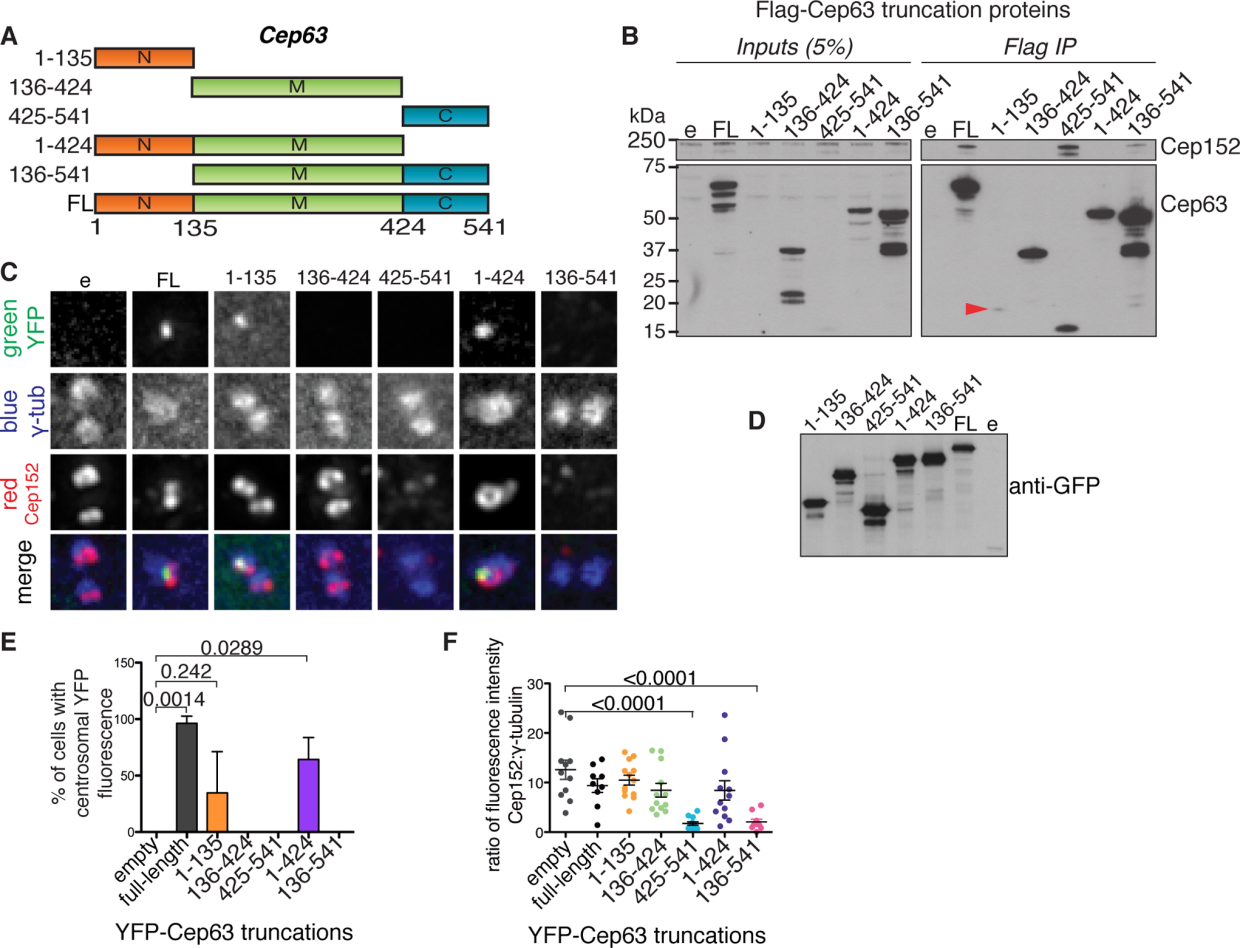
Cep63 N-terminus is required for centrosomal localisation of Cep63 and Cep152. (A) Diagram of Cep63 full length and truncation proteins used in the following experiments. Numbers indicate amino acid positions. (B) Cep63 C-terminus interacts with Cep152. Expression of Flag-Cep63 (FL), truncation proteins, or Flag-empty vector control (e) was induced in 293 FlpIn TREX cell lines by incubation with 2 µg/ml doxycycline for 72 hours, then proteins were immunoprecipitated using anti-Flag resin. Western blots show endogenous Cep152, detected by anti-Cep152 (Bethyl), and Cep63 truncations detected by anti-Cep63 (Millipore). Inputs are 5% of the lysate used for Flag IP. Red arrowhead points to Cep63 truncation 1–135 present in the Flag IP. (C-F) U2OS cells were transfected with YFP-Cep63 (FL), truncation proteins, or YFP-empty vector (e) for 48 hours. (C) Cells were stained with anti-Cep152 (red) and γ-tubulin (blue) antibodies; YFP-tagged proteins were detected by direct fluorescence (green). Scale bar 1 µm. (D) Whole cell lysates from (C) were analysed by Western blot with anti-GFP antibodies to visualise YFP-tagged proteins. (E) The localisation of YFP-Cep63 proteins to the centrosome was scored in 3 independent experiments, n >10. (F) Overexpression of Cep63 425–541 and 136–541 deplete Cep152 from the centrosome. The ratio of Cep152 to γ-tubulin fluorescence intensities at the centrosome was measured for multiple cells from the experiment shown in (C), n >10.

To identify the regions of Cep63 and Cep152 that are required for their interaction and centrosomal localisation, we generated truncated versions of each protein (Figures 1A and 2A). Pull downs of MBP-Cep63 from lysates of 293 HEK cells expressing GFP-tagged Cep152 full length (FL), N-terminal half (amino acids 1–803), or C-terminal half (amino acids 804–1654) proteins demonstrated an interaction between MBP-Cep63 and Cep152 804–1654, as well as the full length protein (Figure 1B). Interestingly, GFP-Cep152 804–1654 was necessary and sufficient for centrosomal localisation (Figure 1C), supporting previous conclusions that the region required for centrosome targeting is within amino acids 1045–1290 [7], [10]. These data indicate that the interaction between Cep63 and Cep152 is required for the localisation of Cep152 to the centrosome, consistent with previous data showing that Cep63 recruits Cep152 to the centrosome [24], [30].

In order to map the region of Cep63 required for interaction with Cep152, we performed Flag IPs from lysates of 293 HEK cell lines expressing inducible Flag-tagged Cep63 full-length protein (FL) or Cep63 truncations and Western blotting to detect endogenous Cep152 (Figure 2B). We found that the Cep63 C-terminal region (amino acids 425–541) was necessary and sufficient for the interaction with Cep152. This finding supports the conclusion that Cep63 isoform a interacts with Cep152 through its C-terminal region (amino acids 382–703 [24]), and narrows down the interaction site further. This region of Cep63 isoform b (425–541) is present in all of the four isoforms of Cep63, all of which are expressed at the mRNA level in U2OS cells, suggesting that all isoforms are able to interact with Cep152 [33].

To determine the region of Cep63 required for centrosomal localisation, YFP-Cep63 full length (FL) protein and truncations were expressed in U2OS cells (Figure 2C–F). YFP-Cep63 FL, as well as truncations containing the N-terminal region of Cep63 (amino acids 1–135), localised to the centrosome (identified by γ-tubulin and Cep152 immunofluorescence). Note that localisation of Cep63 1–135 to the centrosome was less robust (lower percentage of cells with centrosomal localisation, Figure 2E) than 1–424 or full-length Cep63 proteins. Thus, we conclude that the N-terminal region of Cep63 is required for its centrosomal localisation, but that the central region promotes a more robust recruitment or retention of Cep63 to the centrosome. Although Cep63 1–424, which did not interact with Cep152, was able to localise to the centrosome we cannot rule out the possibility that the recruitment of Cep63 1–424 could be influenced by endogenous Cep63-Cep152 complex present in these experiments. Indeed, the small increase in the incidence of centrosomal localisation between Cep63 1–424 and full-length Cep63, suggests that the Cep152 interaction promotes more efficient centrosomal recruitment or retention (Figure 2E).

Intriguingly, we observed a significant decrease of centrosomal Cep152 staining upon overexpression of YFP-Cep63 truncations containing the C-terminal Cep152 interacting region, but lacking the N-terminal centrosome localisation domain (truncations 425–541 and 136–541, Figure 2C and F). Thus, the C-terminal Cep152-interacting domain of Cep63 depletes Cep152 from the centrosome when overexpressed. Neither Cep63 425–541 nor 136–541, showed any centrosomal localisation, indicating that recruitment of the Cep63-Cep152 complex to the centrosome requires the presence of the N-terminal region of Cep63.

Together, these data led us to conclude that Cep63 and Cep152 can interact directly and independently of the centrosome, and that a Cep63–Cep152 complex is recruited to the centrosome as a unit, through the N-terminus of Cep63.

### Cep63 Ensures Efficient Centriole Duplication in Mammalian Cells

Cep63 is required for efficient centriole duplication in chicken DT40 cells, and Cep152 is required for centriole duplication in flies and humans [8], [10], [20], [24], [34]. Furthermore, the localisation of Cep152 is dependent on Cep63 in both chicken DT40 cells and in human cell lines [24], [30], but whether mammalian Cep63 is required for centriole duplication has not been addressed directly.

We investigated the impact of Cep63 depletion on the process of centriole duplication in U2OS cells using Cep63 RNAi and immunofluorescence to detect centrin, a distal centriole marker [35]. Importantly, Cep63 RNAi led to a dramatic reduction in Cep63 fluorescence at the centrosome, to between 1 and 26% of Cep63 fluorescence in control centrosomes (Figure S2). We verified the depletion of Cep63 by immunofluorescence, as we were unable to detect Cep63 by Western blotting of whole cell lysates. Cep63 was detectable by Western blotting only when enriched by immunoprecipitation of Flag-Cep152 (Figure S1B) or immunoprecipitation using the anti-Cep63 antibody (Figure S3B) indicating that Cep63 is likely a protein of very low abundance in the cytoplasm.

**Figure 3:**
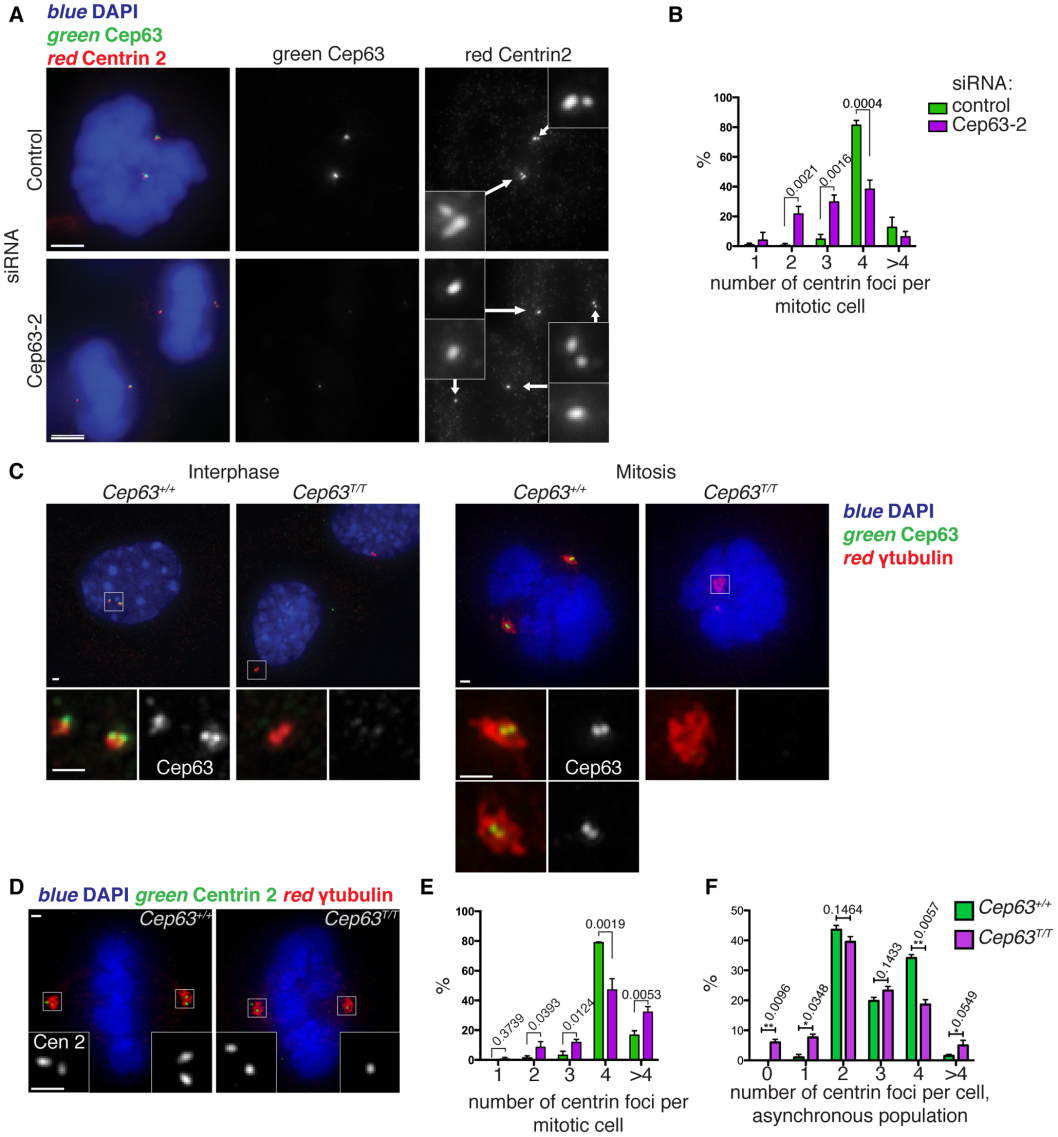
Cep63 is needed for efficient centriole duplication in human and mouse cells. (A-B) Cep63 depletion by RNA interference (RNAi) led to decreased centriole numbers in U2OS cells. Cells treated with control or Cep63 (Cep63-2) short interfering RNAs (siRNAs) for 4 days were stained with anti-centrin 2 (red) and anti-Cep63 (green) antibodies, plus DAPI (blue) to visualise DNA. Small panels show 3 times enlargements of the centrosome region with centrin 2 staining. Scale bar is 5 µm. (B) The number of centrin foci per mitotic cell was scored in three independent experiments, n>60. (C) Cep63 homozygous gene-trap mouse embryonic fibroblasts (MEFs) are devoid of Cep63 protein. Primary MEFs, at passage 3, were stained with anti-Cep63 (green) and anti-γ-tubulin antibodies (red), and DAPI (blue) to detect centrosomes and DNA, respectively. Representative images are shown of interphase and mitotic cells from two cell lines derived from littermates, one homozygous wild type (*Cep63^+/+^*) and the other homozygous gene-trap (*Cep63^T/T^*). Scale bars 1 µm. (D-F) *Cep63^T/T^* MEFs have reduced centriole numbers. (D) Primary MEFs, passage 3, were stained with anti-centrin2 (green) and anti-γ-tubulin (red) antibodies and DAPI. Scale bars 1 µm. (E) Centrin foci were scored in mitotic cells from 3 different cell lines for each genotype at passage 3, 40<n<100. (F) Centrin foci were scored in all cells (all cell cycle stages) in passage 1 primary MEF cell lines from 3 *Cep63^+/+^* and 3 *Cep63^T/T^* embryos, n >100.

In order to eliminate the effect of cell cycle changes on centriole number, centrin foci were counted in mitotic cells, which should have 4 foci (2 per centrosome). Cep63 RNAi led to a significant increase in mitotic cells with fewer than 4 centrin foci, indicating a defect in centriole duplication (Figure 3A–B). Consistent with previous results, depletion of Cep152 led to a similar phenotype (Figure S4A–B) [7], [8], [10], [34]. Cep152 depletion was verified by immunofluorescence and by Western blotting (Figure S2).

We sought to confirm these results in primary mouse embryonic fibroblasts (MEFs). We obtained MEFs from mouse embryos homozygous for a Cep63 allele interrupted by a genetrap (*Cep63^T/T^*) insertion between exons 1 and 2. PCR analysis, Western blotting and immunofluorescence confirmed that no Cep63 mRNA or protein was detectable in these cells (Figure 3C and Figure S3). Centrioles were counted in primary MEFs between passage 1 and 3 using centrin as a marker of centrioles and γ-tubulin to mark the position of the centrosome(s). *Cep63^T/T^* MEF cultures showed a defect in centriole duplication: 20% of mitotic cells contained fewer than four centrin foci (Figure 3D–E). Upon counting centrin foci in asynchronous MEF populations, a significant increase in cells containing less than 2 centrin foci was observed in *Cep63^T/T^* cells compared to wild type controls (Figure 3F). Note that cells should contain a minimum of two centrioles, as newly formed G1 cells should inherit a pair of centrioles. Importantly, Cep63 depleted mitotic cells often contained only one centriole per centrosome, which indicates that there is a problem in the process of procentriole formation, rather than a problem in centriole disengagement, which is a prerequisite for centriole duplication (Figure 3) [36].

**Figure 4:**
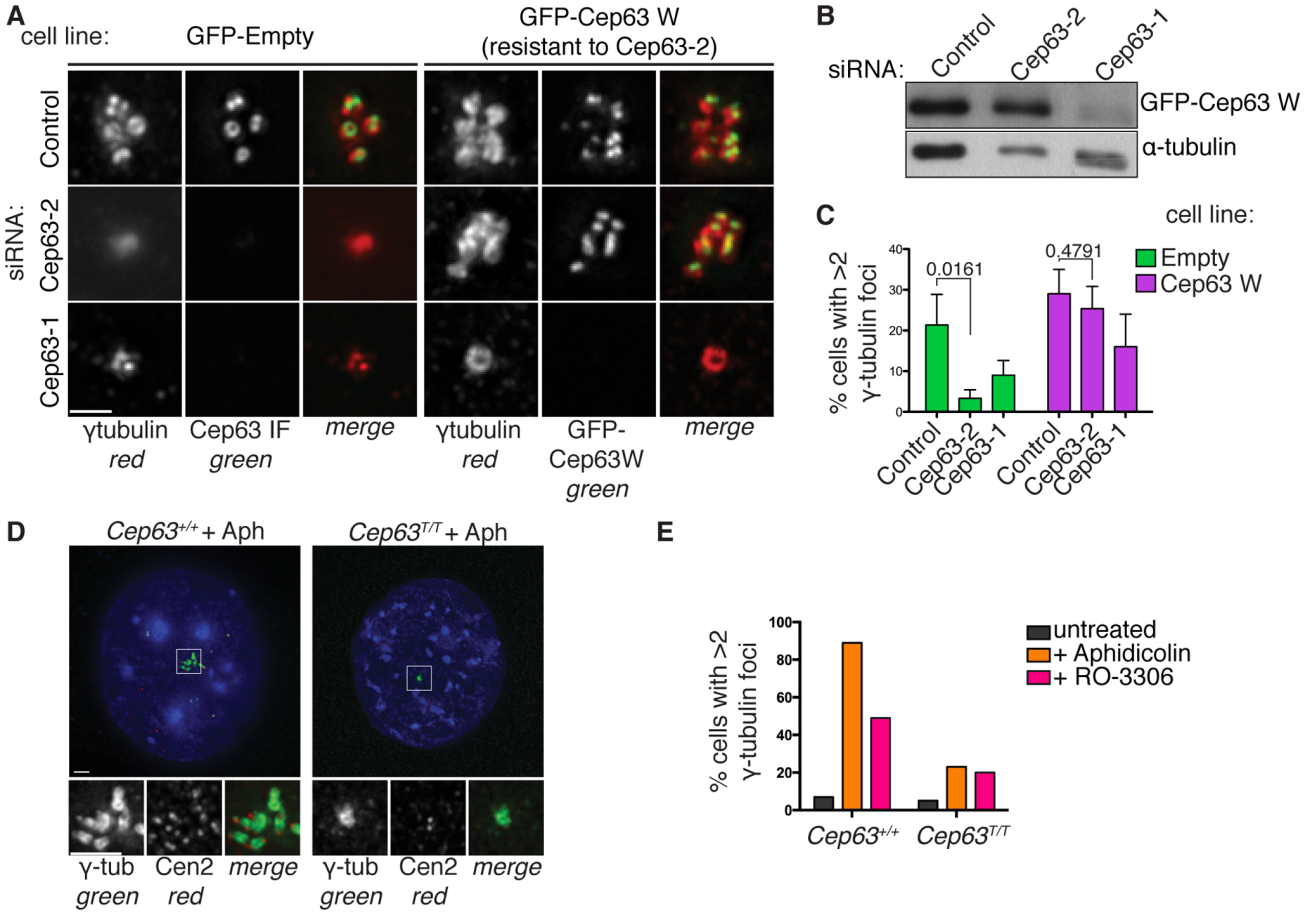
Cep63 and Cep152 are required for centrosome reduplication in mammalian cells. (A) U2OS cells stably expressing GFP or GFP-Cep63 W (resistant to siRNA Cep63-2) were treated with Cep63 siRNAs 1 or 2 and incubated with 1.9 µg/ml aphidicolin (Aph) for 72 hours. GFP cells were stained with anti-Cep63 (green) and γ-tubulin (red). GFP-Cep63 W cells were stained with anti-γ-tubulin (red) and GFP-Cep63 W was detected by direct fluorescence. Scale bar 1 µm. (B) U2OS GFP-Cep63 W cell lysates were analysed by Western blotting with anti-GFP to detect GFP-Cep63 W and α-tubulin antibodies, used as a loading control. (C) Cells with greater than 2 γ-tubulin foci were recorded in 3 independent experiments, n = 150. T-test p values are indicated for comparison of control and siRNA Cep63-2 in both cell lines. (D-E) Cep63 homozygous gene-trap MEFs (*Cep63^T/T^*) show reduced centrosome reduplication induced by aphidicolin or Cdk1 inhibitor. (D) *Cep63^+/+^* or *Cep63^T/T^* 3T3 immortalised MEFs incubated with aphidicolin (2 µg/ml) for 72 hours, stained with anti- γ-tubulin antibodies (green), centrin 3 antibodies (red) and DAPI (blue). Scale bar 5 µm. (H) Quantification of *Cep63^+/+^* and *Cep63^T/T^* MEFs with greater than 2 γ-tubulin foci in untreated, Cdk1 inhibitor (RO-3306, 10 µM), or aphidicolin treated populations, n>150.

Next, we examined centrosome reduplication which occurs in certain cell types upon prolonged cell cycle arrest induced by DNA replication inhibitors [37]. Depletion of Cep152 has been shown to reduce centrosome reduplication in U2OS cells [7]. On the other hand, DT40 CEP63 knock-out cells show centrosome reduplication at levels comparable to wild type when incubated with aphidicolin [24]. We took advantage of the centrosome reduplication observed in U2OS cells and used Cep152 RNAi as a positive control, which led to a reduction in centrosome amplification (Figure S4C–D), as previously reported [7]. Cep63 RNAi caused a similar inhibition of centrosome amplification (Figure 4A–C, and Figure S4C–D). Importantly, the decrease in centrosome amplification was observed with two different Cep63 siRNAs, and centrosome amplification was restored upon expression of an RNAi resistant copy of GFP-Cep63 (GFP-Cep63 W, Figure 4A–C).

We also observed centriole reduplication in MEFs immortalised according to the 3T3 protocol and incubated with either aphidicolin or the Cdk1 inhibitor (RO-3306) for 72 hours. Consistent with the data from human cells, centrosome amplification was greatly reduced in *Cep63^T/T^* MEFs compared to wild type controls (Figure 4D–E). Centrosome amplification induced by incubation with DNA replication stalling agents is caused by a prolonged G2-like phase and is dependent on Plk1 and APC/C activity [38]–[40]. Prevention of mitotic entry by inhibition of Cdk1 results in a prolonged G2 phase and Plk1 becomes active, resulting in centrosome amplification in Chinese hamster ovary and chicken DT40 cells [41]. We show here that the same phenomenon also occurs in MEFs, and that centrosome reduplication is significantly reduced in the absence of Cep63. Although chicken CEP63 is required for centriole duplication in unperturbed DT40 cells, aphidicolin induced centrosome amplification can still occur in in the absence of CEP63 [24], which may indicate a species or cell type dependent difference in Cep63 function.

Although centriole duplication defects were observed in U2OS cells depleted of Cep63 and in *Cep63^T/T^* MEFs, both experimental approaches demonstrated that lack of Cep63 led to impairment of centriole duplication, but not complete inhibition. Therefore, we conclude that Cep63 is not required for centriole biogenesis *per se*, but that it is required for efficient and timely centriole duplication, consistent with studies in chicken DT40 Cep63 knock-out cells [24].

### Cep63 and Cep152 Function Downstream of the Conserved Centriole Duplication Factor Cep192

**Figure 5:**
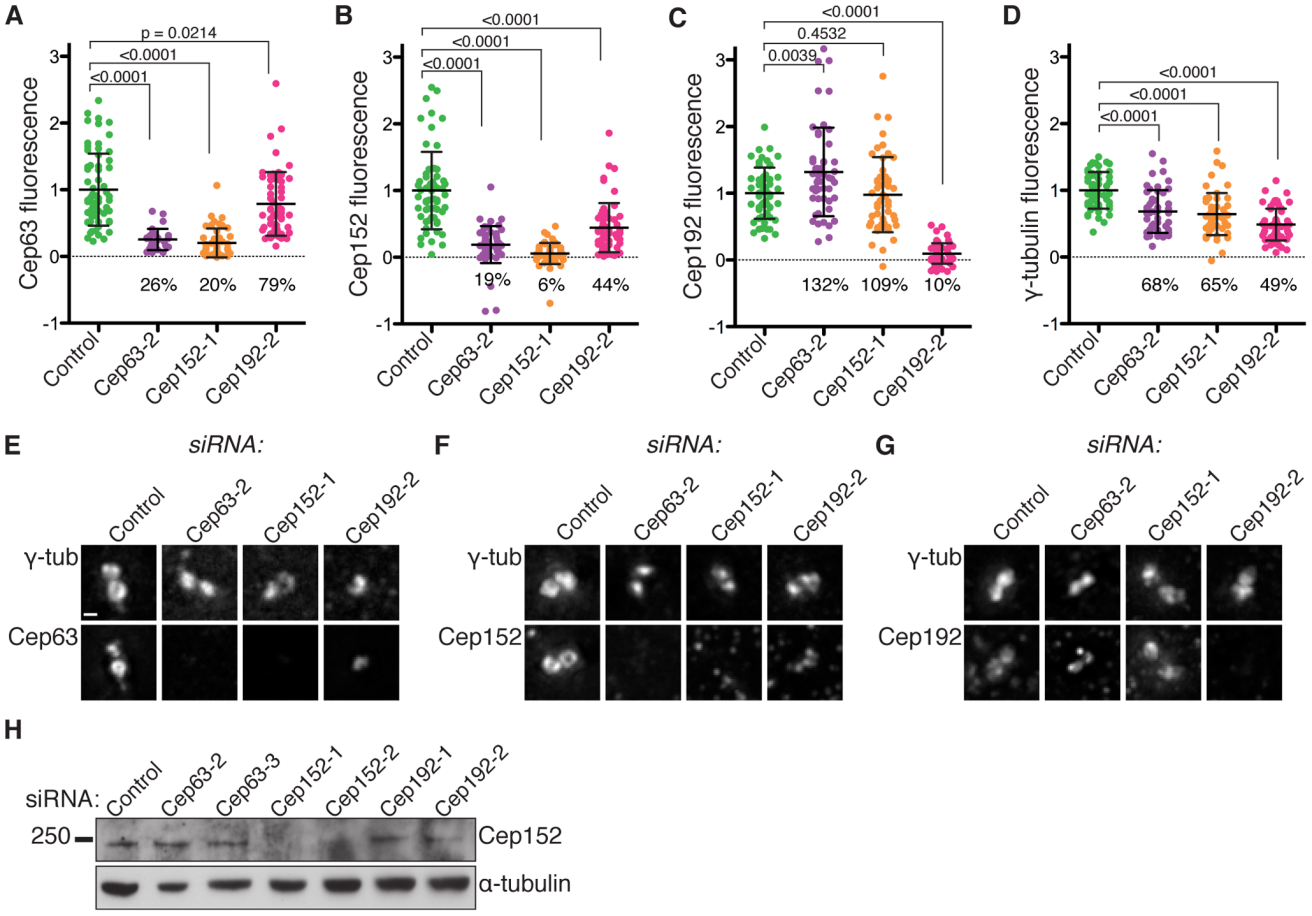
Cep63-Cep152 centrosomal recruitment is downstream of Cep192. (A-D) Control RNAi or RNAi of Cep63, Cep152, or Cep192, was carried out for 4 days in U2OS cells, followed by immunofluorescence on replicate samples, with anti- γ-tubulin and anti-Cep63 (A), Cep152 (B), or Cep192 (C) antibodies. Fluorescence intensity of Cep63, Cep152, Cep192, and γ-tubulin at the centrosome were measured (graphs A-D). All intensity measurements were normalised to the mean of the control population and p values are indicated above (students’ t-test). (E-G) Images of γ-tubulin and Cep63, Cep152, or Cep192 immunofluorescence at the centrosome from the experiment shown in A-D. Scale bar 1 µm. (H) Western blots of whole cell lysates from U2OS cells used in experiments (A-G) using anti-Cep152 (Bethyl) and α-tubulin antibodies.

A potential role of Cep63-Cep152 in centriole duplication is to promote recruitment of PCM and the proteins required for centriole duplication. Both of these processes require Cep192 [6], whose orthologue, SPD-2, functions upstream of the known centriole duplication components in *C. elegans* [5]. In order to determine the functional relationship of Cep63 and Cep152 with Cep192, we depleted each protein by RNAi and analysed centrosomal protein levels by immunofluorescence (Figure 5). Western blotting analysis indicated that Cep152 total protein levels were efficiently reduced after treatment with Cep152 siRNA, and unaffected by RNAi of Cep63 or Cep192 (Figure 5H). While Cep63 or Cep152 RNAi efficiently depletes both Cep63 and Cep152 from the centrosome (Figure 5A,B,E and F), centrosomal levels of Cep192 were unaffected (Figure 5C and G). On the other hand, Cep192 RNAi effectively depleted Cep192 levels at the centrosome (Figure 5C and G) and significantly reduced the centrosomal levels of both Cep152 and Cep63 (Figure 5A–B and E-F).

The reduction of Cep63 and Cep152 at the centrosome after depletion of Cep192 was mild compared to the reduction after depletion of Cep63 or Cep152. Thus we conclude that Cep192 is required for full recruitment of Cep63 and Cep152 to the centrosome, but that it this is likely through an indirect mechanism. Conversely, Cep192 is not dependent on Cep63–Cep152 for centrosomal localisation.

These data also add to recent findings that describe the role of Cep152 in centrosomal localisation of Cep63. Others recently observed reduction of Cep63 centrosomal fluorescence by 60–80% after Cep152 depletion, and our data shows a reduction of 80–95% after Cep152 RNAi, depending on the extent of Cep152 depletion (Figure 5A and E, and Figure S2A) [30]. Since we were unable to detect total Cep63 protein levels by Western blotting of whole cell lysates, Cep152 RNAi was repeated in U2OS cells expressing GFP-Cep63 in order to establish whether Cep152 RNAi specifically affects Cep63 localisation (Figure S2C–D). Centrosomal localisation of GFP-Cep63 was abolished after Cep152 RNAi, but total protein levels were unaffected, confirming that it is specifically the localisation of Cep63 that is dependent on Cep152.

### Depletion of Cep63 and Cep152 Leads to Delayed Recruitment of SAS-6

**Figure 6:**
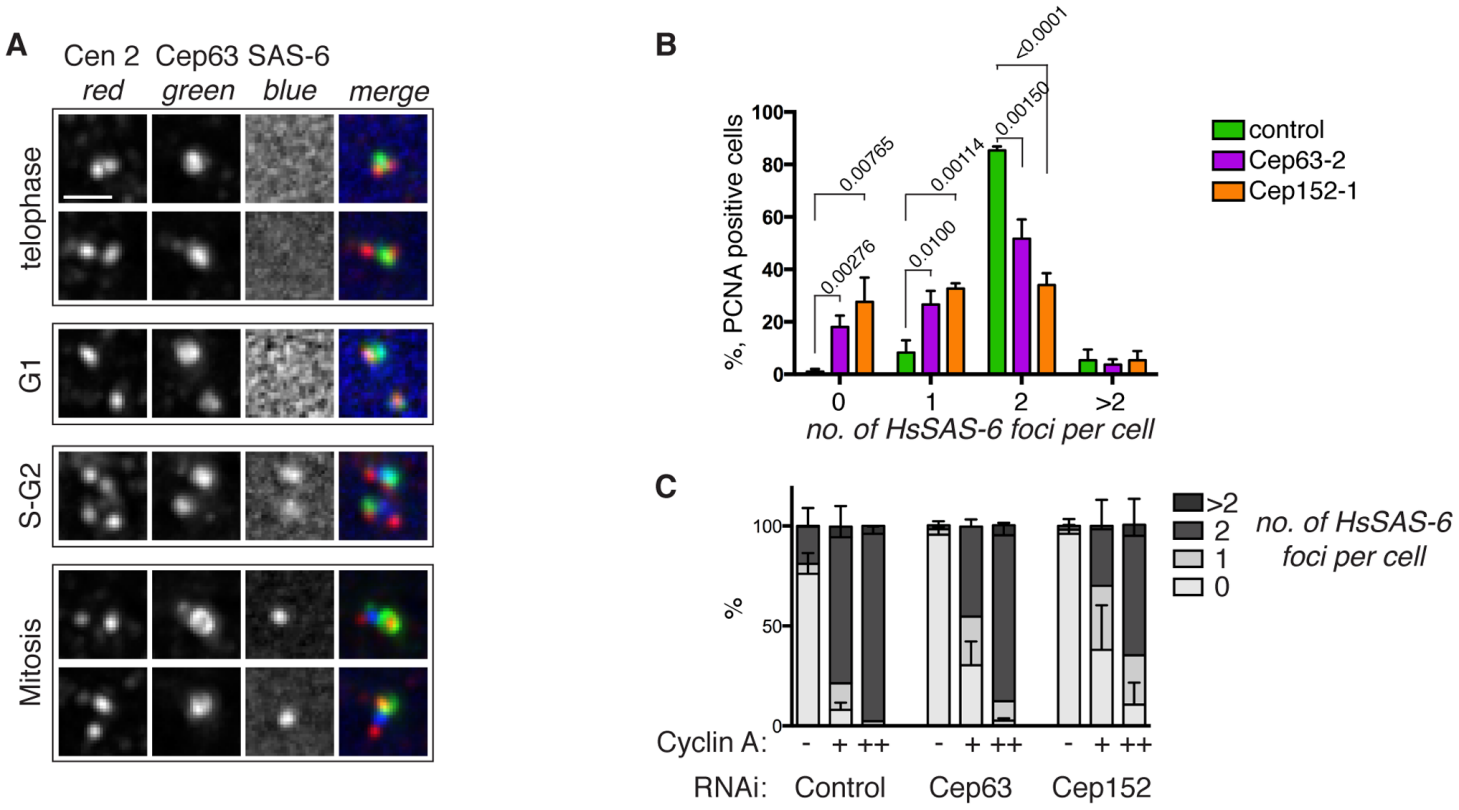
Lack of centrosomal Cep63–Cep152 causes a delay in HsSAS-6 recruitment. (A) Cep63 is present at the PCM before HsSAS-6 recruitment. Telophase, G1 phase, S or G2 phase and mitotic HeLa cells, as indicated, were stained with anti-centrin 2 (red), Cep63 (green), and HsSAS-6 (blue) antibodies. Centrosomes from each cell are shown. Scale bar 1 µm. (B) HsSAS-6 foci were counted in U2OS cells with nuclear PCNA foci (a marker of DNA replication) after 96 hours RNAi as indicated, n >150, 3 experiments. P values from a students’ t-test are indicated on the graph. (C) U2OS cells were stained with anti-Cyclin A and HsSAS-6 antibodies after control, Cep63, or Cep152 RNAi. Cyclin A status, negative (-, early G1 phase), dull (+, G1-S), or bright (++, S-G2), and the number of HsSAS-6 foci (0,1,2,>2) were scored in asynchronous populations in three independent experiments, n >150.

Total HsSAS-6 protein levels are reduced upon mitotic exit, then as cellular HsSAS-6 levels increase, it is recruited to disengaged centrioles during G1 and S phase [42]. Observation of disengaged centrioles (marked by centrin 2) that are Cep63 positive, but with no detectable HsSAS-6, indicates that Cep63 was present in the PCM surrounding both centrioles prior to HsSAS-6 recruitment to the newly forming procentrioles (Figure 6A).

In order to determine if Cep63 and Cep152 influence HsSAS-6 recruitment, the first step of procentriole formation, we analysed HsSAS-6 localisation after depletion of Cep63 or Cep152 by RNAi (Figure 6B). HsSAS-6 foci were counted in cells with nuclear PCNA foci, a marker of DNA replication [43]. The majority of cells treated with control siRNA contained two HsSAS-6 foci as expected, with only 9% containing fewer. However, in cells depleted of Cep63 or Cep152, the majority of the S phase populations contained less than 2 HsSAS-6 foci, 35% and 60% respectively, indicating that HsSAS-6 recruitment is severely delayed or inhibited in the absence of the Cep63–Cep152 complex at the centrosome.

Next, we analysed HsSAS-6 foci in asynchronous U2OS cells treated with Cep63 or Cep152 RNAi (Figure 6C). Cells were categorised by cell cycle stage using Cyclin A immunofluorescence (Cyclin A negative, early G1; dull Cyclin A, G1-S phase; bright Cyclin A, S-G2 phase) [44], and HsSAS-6 foci were counted. In cells treated with control siRNA, most Cyclin A negative cells were HsSAS-6 negative, as expected, although a small proportion already had two HsSAS-6 foci visible. Upon transition to early S phase, the proportion of cells with HsSAS-6 foci increased until the majority of cells contained two HsSAS-6 foci in S and G2 phases. However, depletion of Cep63 or Cep152 resulted in an increase in G1-S and S-G2 phase cells with no visible HsSAS-6 foci, or only one visible focus (Figure 6C). Similarly, in asynchronous MEFs, single SAS-6 foci (indicating recruitment of SAS-6 to one centrosome, but not the other) were observed in *Cep63^T/T^* but not wild type controls, which were either SAS-6 negative or contained two SAS-6 foci (Figure S5).

Together, these data indicate that HsSAS-6 recruitment to the centrosome is delayed or in some cases, abolished, in the absence of Cep63 and Cep152. Importantly, the cell cycle stage with the most pronounced defect in HsSAS-6 recruitment is S phase, when HsSAS-6 is first recruited to the centrosome and when it plays its role in centriole biogenesis by forming the cartwheel structure at the base of the procentriole [4], [42].

Consistent with a role for the Cep63–Cep152 complex in the recruitment of essential centriole duplication factors to the PCM, a slight decrease in γ-tubulin area and fluorescence was observed in U2OS cells after RNAi depletion of Cep63 or Cep152 (Figure S6A–B, and Figure 5D) and a decrease in γ-tubulin area was observed in *Cep63^T/T^* primary MEFs compared to a *Cep63^+/+^* littermate control (Figure S6C–D). This has previously been observed for Cep152 [8]. A more pronounced decrease in centrosomal γ-tubulin fluorescence intensity was observed after depletion of Cep192 from the centrosome, as previously reported (Figure 5D) [6]. Collectively, these data suggest that the Cep63–Cep152 complex promotes the recruitment of centriole biogenesis proteins to the pericentriolar material in order to ensure SAS-6 recruitment and procentriole formation.

## Discussion

Previously, Cep63 was shown to promote the recruitment of Cep152 to the centrosome and to form a complex with both Cep152 and Cep57 [24], [30]. The work reported here further extends our knowledge of the nature of the Cep63–Cep152 complex and the requirements for its localisation to the centrosome. We have also described a previously uncharacterised centriole duplication phenotype observed upon Cep63 depletion in mammalian cells and provided evidence that the Cep63–Cep152 complex acts upstream of SAS-6 recruitment to promote procentriole formation.

Centriole duplication is dependent on the kinase activity and centrosomal localisation of Plk4, and its efficient centrosomal localisation requires Cep152 [7]–[10]. Cep152 likely plays additional roles downstream of Plk4 as it can be phosphorylated by Plk4 *in vitro* and is essential for CPAP recruitment *via* a direct interaction [7], [8], [10].

Our data indicates that Cep63 interacts with Cep152 directly and independently of centrosomal localisation. As we observed only low levels of centrosomal Cep63 upon depletion of Cep152, and vice versa, it is likely that they cannot localise or efficiently accumulate at the centrosome independently of one other. Furthermore, we found that the N-terminal region 1–135 of Cep63 was required for localisation of the Cep63–Cep152 complex to the centrosome. An intriguing possibility is that cellular localisation could be regulated at the level of Cep63–Cep152 complex formation through post-translational modifications.

Cep63 deficient mitotic cells often contained fewer than four centrin foci and among these were cells with only one centrin focus per spindle pole, either at one pole or both, indicating that centrioles disengaged but failed to duplicate. Thus, we conclude that there is a specific effect on centriole biogenesis, rather than an indirect effect on centriole duplication due to failure of the centrioles to disengage at the end of mitosis, which would render them unable to duplicate in the following S phase. Similar centriole duplication defects were observed upon depletion of either Cep63 or Cep152 by RNAi, consistent with the observation that both Cep63 and Cep152 are depleted from the centrosome to similar extents under each of these conditions, and consistent with the idea that they function together.

Cep63 depletion resulted in inefficient centriole duplication such that centriole duplication could occur, but that centrioles were not reliably duplicated every cell cycle. The heterogeneity of this centriole duplication phenotype has also been reported in Cep63 knock-out chicken DT40 cells [24], and could be due to intra-cell line heterogeneity. One could imagine that differences in cell cycle timing could result in phenotypic heterogeneity due to differences in the time allowed for SAS-6 recruitment to the centrosome. We hypothesise that in the absence of Cep63–Cep152, centriole duplication factor recruitment to the PCM, including SAS-6, is less efficient. Therefore, some centrosomes may not recruit the threshold level of SAS-6 required to support procentriole formation in the time taken for the cell to progress through interphase, while others may acquire sufficient SAS-6 in time. Indeed, the amount of HsSAS-6 at centrioles is crucial for determining the state of procentriole formation [42].

Previous studies have reported conflicting data regarding the role of Cep152 in procentriole formation with respect to HsSAS-6 recruitment [7], [10]. Our work demonstrates, using both RNAi of Cep63 or Cep152 and a genetic approach to block Cep63 expression, that efficient SAS-6 recruitment in S phase requires the Cep63–Cep152 complex. An additional role for Cep63–Cep152 in recruiting factors downstream of SAS-6 is also possible. In fact, Cep152 interacts with CPAP and is required for its recruitment [7], [8].

Cep192 is required for centriole duplication in mammalian cells, but its function has yet to be elucidated [6]. We found that Cep192 localised to the centrosome independently of Cep63 and Cep152, but that recruitment of both Cep63 and Cep152 was slightly impaired in the absence of Cep192. Thus, we propose that Cep63 and Cep152 are recruited downstream of Cep192 but that this is likely an indirect effect, as some Cep192 independent recruitment of Cep63 and Cep152 does occur. This is consistent with recent sub diffraction-resolution fluorescence imaging data showing that the toroid shape formed by Cep192, around the mother centriole within the PCM, has a smaller diameter than that formed by Cep152, indicating that the recruitment of Cep152 is likely to occur after that of Cep192 [31], [45]. Our data indicate that Cep192 may have an asymmetric influence on Cep63 and Cep152 localisation. However, further work is needed to clarify the relationship between Cep192 and the Cep63–Cep152 complex and to identify the role of additional players in Cep63–Cep152 centrosomal recruitment, such as Cep57 [30].

Collectively, our data, and that of others, indicates that Cep63 and Cep152 form a complex that plays a positive role in the regulation of centriole duplication. We propose that in the absence of Cep63–Cep152, the threshold level of centriole duplication proteins is not always reached, leading to stochastic defects in centriole configurations as has also been observed in DT40 cell lines [24].

As both Cep63 and Cep152 are mutated in hereditary human microcephaly, further analysis of their functional interactions, as well as the detailed characterisation of mice lacking Cep63, will be valuable for understanding their role in centriole duplication and its relation to the aetiology of microcephaly.

## Supporting Information

Figure S1 **Cep63 and Cep152 interact.** (A) YFP-Cep63 interacts with Flag-Cep152 in 293 HEK cells. Cells constitutively expressing YFP-Cep63 were transfected with either Flag empty vector (-) or Flag-Cep152 (+) and proteins were immuno-precipitated using anti-Flag antibody coupled beads. Input shows 10% of the whole cell lysate used for IP. Immuno-precipitated proteins were visualised by Western blotting with anti-Flag or anti-GFP antibodies. (B) Flag IP from 293 HEK cells stably expressing Flag-empty vector or Flag-Cep152 using 5 mg whole cell lysate; inputs show 100 µg whole cell lysate. Endogenous and Flag-tagged Cep152 were detected by Western blotting with anti-Cep152 antibodies (Bethyl) and endogenous Cep63 was detected using anti-Cep63 (Millipore). (C) *Xenopus laevis Cep63* (XCep63) interacts with *Xenopus laevis* Cep152 (XCep152) in the absence of centrosomes. Amylose resin bound to MBP or MBP-XCep152 was incubated with *Xenopus laevis* cytostatic factor arrested egg extracts containing *in vitro* transcribed/translated XCep63 or Luciferase labelled with ^35^S methionine. Proteins were eluted by boiling in Laemmli buffer, analysed by SDS-PAGE, and detected by autoradiography. The same experiment was carried out in the absence of egg extract, using EB buffer (50 mM HEPES pH 7.5, 100 mM KCl, 2.5 mM MgCl_2_) as a replacement (no extract).
(TIF)

Figure S2 **Cep63 and Cep152 are required for efficient centriole duplication and reduplication in human cells.** (A) U2OS cells after Control, Cep63, or Cep152 RNAi for 96 hours, stained with anti-Centrin 2 (green) and γ-tubulin (red) antibodies and DAPI (blue). Lower panels show 3-fold enlargements of Centrin 2 staining at the centrosomes (boxed regions). Scale bar 5 µm. (B) Quantification of Centrin foci number in mitotic U2OS cells after Control, Cep63, or Cep152 RNAi from 3 independent experiments, n>20. Significant differences between the percentage of cells with fewer than 4 Centrin foci are indicated with p values calculated by a students’ t-test. (C) U2OS cells depleted of Cep63 or Cep152 by RNAi were incubated with 1.9 µg/ml aphidicolin for 72 hours. Pictures show γ-tubulin immunofluorescence (green, white in inserts) and DAPI (blue). Scale bar 5 µm. (D) Cells with more than 2 γ-tubulin foci were counted in 3 independent experiments, n = 150.
(TIF)

Figure S3 **Cep63 gene-trap homozygous MEFs lack Cep63 mRNA and protein.** (A) Messenger RNA from *Cep63^+/+^* and *Cep63^T/T^* MEFs was analysed by reverse transcription-PCR using primers located within the gene-trap or within different Cep63 exons, as indicated. (B) Western blot of whole cell lysates (100 µg) and immuno-precipitates from 2 mg whole cell lysates of *Cep63^+/+^* and *Cep63^T/T^* cell lines with pre-immune IgG (control), or two different Cep63 specific purified antibodies, M (Millipore) and P (Protein Tech Group). The Cep63 Millipore antibody was used for Western blotting. Arrow indicates a Cep63 specific band that is present in *Cep63^+/+^*, but not *Cep63^T/T^* MEFs.
(TIF)

Figure S4 **Cep63 and Cep152 are dependent on each other for centrosomal localisation.** (A-B) Cep63 and Cep152 are dependent on each other for centrosomal localisation. (A) Control, Cep63 (63-2 and 63-3), or Cep152 (152-1 and 152-2) RNAi was carried out over 4 days in U2OS cells and the fluorescence intensities of Cep63 (left), Cep152 (middle), and γ-tubulin (right) were measured at the centrosome in multiple cells (n>25) in 3 experiments. The graphs show the mean fluorescence intensities normalised to the mean of the control population and the standard deviation, and p values are indicated above (*** denotes p<0.0001). Images of centrosomes from these cells are shown, from cells stained with anti-Cep63 (left) or Cep152 (right) in green and γ-tubulin (red). (B) Cep63 RNAi does not affect total levels of Cep152 protein. Western blot of whole cell lysates of U2OS after 4 days of RNAi treatment with the siRNAs indicated, showing endogenous Cep152 and α-tubulin as a loading control. (C) Quantification of GFP-Cep63 fluorescence intensity (GFP direct fluorescence) at the centrosomes of U2OS cells expressing GFP-Cep63 and transfected with Control, Cep63 (63-3), or Cep152 (152-1) siRNAs, n = 30. *** Indicates a p value of <0.0001 calculated using a students’ t-test. Representative pictures of centrosomes from these cells stained with anti-Cep152 (red) and γ-tubulin (blue) antibodies are shown in the right hand panel. GFP fluorescence is shown in green. Scale bar 1 µm. (D) Cep152 RNAi does not affect total levels of GFP-Cep63 protein. Western blot of whole cell lysates from cells used in (C) using anti-Cep152, GFP, and α-tubulin antibodies.
(TIF)

Figure S5 **Impaired SAS-6 recruitment in Cep63 deficient mouse cell lines.** *Cep63^+/+^* or *Cep63^T/T^* MEF cell lines, immortalised by SV40 large T antigen expression, were incubated with aphidicolin (2 µg/ml) for 24 hours, then fixed and stained with anti-HsSAS-6 antibodies. The number of SAS-6 foci per cell was counted for three *Cep63^+/+^* cell lines and three *Cep63^T/T^* littermate controls, n >100. The difference in percentage of cells with only one SAS-6 focus was significant (p = 0.0009, students’ t-test).
(TIF)

Figure S6 **Reduced centrosomal γ-tubulin after Cep63–Cep152 depletion.** (A-B) Depletion of Cep63 and Cep152 leads to a slight reduction in PCM size. (A) Images of centrosomes from U2OS cells treated with control, or two different Cep63 (63-2, 63-3) or Cep152 (152-1, 152-2) siRNAs, stained with anti-γ-tubulin and Cep63 or Cep152 antibodies. Scale bar 5 µm. (B) γ-tubulin area was measured in interphase cells, from z-stack projections, using Image J software in number of pixels, n >25. (C-D) *Cep63^T/T^* primary MEFs have a slight reduction in PCM size in both interphase and mitosis. (C) Images of interphase and mitotic *Cep63^+/+^* or *Cep63^T/T^* primary MEFs at passage 4, stained with anti- γ-tubulin (red) and Cep63 (green) antibodies and DAPI (blue). Scale bars are 5 µm for large panels and 1 µm for small panels, which show enlargements of each centrosome. (D) γ-tubulin area was measured in interphase and mitotic cells, from z-stack projections, using Image J software in number of pixels, n >50 for interphase cells and n >43 for mitotic cells.
(TIF)

Supporting Information S1 (PDF)

## References

[1] Gönczy P (2012) Towards a molecular architecture of centriole assembly. Nat Rev Mol Cell Biol 13: 425–435. 22691849 10.1038/nrm3373

[2] Nigg EA, Raff JW (2009) Centrioles, centrosomes, and cilia in health and disease. Cell 139: 663–678. 19914163 10.1016/j.cell.2009.10.036

[3] Nigg EA (2007) Centrosome duplication: of rules and licenses. Trends Cell Biol 17: 215–221. 17383880 10.1016/j.tcb.2007.03.003

[4] Strnad P, Gönczy P (2008) Mechanisms of procentriole formation. Trends Cell Biol 18: 389–396. 18620859 10.1016/j.tcb.2008.06.004

[5] Delattre M, Canard C, Gönczy P (2006) Sequential protein recruitment in C. elegans centriole formation. Curr Biol 16: 1844–1849. 16979563 10.1016/j.cub.2006.07.059

[6] Zhu F, Lawo S, Bird A, Pinchev D, Ralph A, et al (2008) The mammalian SPD-2 ortholog Cep192 regulates centrosome biogenesis. Curr Biol 18: 136–141. 18207742 10.1016/j.cub.2007.12.055

[7] Cizmecioglu O, Arnold M, Bahtz R, Settele F, Ehret L, et al (2010) Cep152 acts as a scaffold for recruitment of Plk4 and CPAP to the centrosome. J Cell Biol 191: 731–739. 21059844 10.1083/jcb.201007107 PMC2983070

[8] Dzhindzhev NS, Yu QD, Weiskopf K, Tzolovsky G, Cunha-Ferreira I, et al (2010) Asterless is a scaffold for the onset of centriole assembly. Nature 467: 714–718. 20852615 10.1038/nature09445

[9] Habedanck R, Stierhof YD, Wilkinson CJ, Nigg EA (2005) The Polo kinase Plk4 functions in centriole duplication. Nat Cell Biol 7: 1140–1146. 16244668 10.1038/ncb1320

[10] Hatch EM, Kulukian A, Holland AJ, Cleveland DW, Stearns T (2010) Cep152 interacts with Plk4 and is required for centriole duplication. J Cell Biol 191: 721–729. 21059850 10.1083/jcb.201006049 PMC2983069

[11] Kleylein-Sohn J, Westendorf J, Le Clech M, Habedanck R, Stierhof YD, et al (2007) Plk4-induced centriole biogenesis in human cells. Dev Cell 13: 190–202. 17681131 10.1016/j.devcel.2007.07.002

[12] Bettencourt-Dias M, Rodrigues-Martins A, Carpenter L, Riparbelli M, Lehmann L, et al (2005) SAK/PLK4 is required for centriole duplication and flagella development. Curr Biol 15: 2199–2207. 16326102 10.1016/j.cub.2005.11.042

[13] Kitagawa D, Vakonakis I, Olieric N, Hilbert M, Keller D, et al (2011) Structural basis of the 9-fold symmetry of centrioles. Cell 144: 364–375. 21277013 10.1016/j.cell.2011.01.008 PMC3089914

[14] van Breugel M, Hirono M, Andreeva A, Yanagisawa HA, Yamaguchi S, et al (2011) Structures of SAS-6 suggest its organization in centrioles. Science 331: 1196–1199. 21273447 10.1126/science.1199325

[15] Pelletier L, O’Toole E, Schwager A, Hyman AA, Muller-Reichert T (2006) Centriole assembly in Caenorhabditis elegans. Nature 444: 619–623. 17136092 10.1038/nature05318

[16] Kitagawa D, Kohlmaier G, Keller D, Strnad P, Balestra FR, et al (2011) Spindle positioning in human cells relies on proper centriole formation and on the microcephaly proteins CPAP and STIL. J Cell Sci 124: 3884–3893. 22100914 10.1242/jcs.089888

[17] Tang CJ, Lin SY, Hsu WB, Lin YN, Wu CT, et al (2011) The human microcephaly protein STIL interacts with CPAP and is required for procentriole formation. EMBO J 30: 4790–4804. 22020124 10.1038/emboj.2011.378 PMC3243611

[18] Vulprecht J, David A, Tibelius A, Castiel A, Konotop G, et al (2012) STIL is required for centriole duplication in human cells. J Cell Sci 125: 1353–1362. 22349705 10.1242/jcs.104109

[19] Lin YC, Chang CW, Hsu WB, Tang CJ, Lin YN, et al (2013) Human microcephaly protein CEP135 binds to hSAS-6 and CPAP, and is required for centriole assembly. EMBO J 32: 1141–1154. 23511974 10.1038/emboj.2013.56 PMC3630357

[20] Guernsey DL, Jiang H, Hussin J, Arnold M, Bouyakdan K, et al (2010) Mutations in centrosomal protein CEP152 in primary microcephaly families linked to MCPH4. Am J Hum Genet 87: 40–51. 20598275 10.1016/j.ajhg.2010.06.003 PMC2896783

[21] Gul A, Hassan MJ, Hussain S, Raza SI, Chishti MS, et al (2006) A novel deletion mutation in CENPJ gene in a Pakistani family with autosomal recessive primary microcephaly. J Hum Genet 51: 760–764. 16900296 10.1007/s10038-006-0017-1

[22] Hussain MS, Baig SM, Neumann S, Nurnberg G, Farooq M, et al (2012) A truncating mutation of CEP135 causes primary microcephaly and disturbed centrosomal function. Am J Hum Genet 90: 871–878. 22521416 10.1016/j.ajhg.2012.03.016 PMC3376485

[23] Kumar A, Girimaji SC, Duvvari MR, Blanton SH (2009) Mutations in STIL, encoding a pericentriolar and centrosomal protein, cause primary microcephaly. Am J Hum Genet 84: 286–290. 19215732 10.1016/j.ajhg.2009.01.017 PMC2668020

[24] Sir JH, Barr AR, Nicholas AK, Carvalho OP, Khurshid M, et al (2011) A primary microcephaly protein complex forms a ring around parental centrioles. Nat Genet 43: 1147–1153. 21983783 10.1038/ng.971 PMC3299569

[25] Fish JL, Kosodo Y, Enard W, Paabo S, Huttner WB (2006) Aspm specifically maintains symmetric proliferative divisions of neuroepithelial cells. Proc Natl Acad Sci U S A 103: 10438–10443. 16798874 10.1073/pnas.0604066103 PMC1502476

[26] Gruber R, Zhou Z, Sukchev M, Joerss T, Frappart PO, et al (2011) MCPH1 regulates the neuroprogenitor division mode by coupling the centrosomal cycle with mitotic entry through the Chk1-Cdc25 pathway. Nat Cell Biol 13: 1325–1334. 21947081 10.1038/ncb2342

[27] Andersen JS, Wilkinson CJ, Mayor T, Mortensen P, Nigg EA, et al (2003) Proteomic characterization of the human centrosome by protein correlation profiling. Nature 426: 570–574. 14654843 10.1038/nature02166

[28] Smith E, Dejsuphong D, Balestrini A, Hampel M, Lenz C, et al (2009) An ATM- and ATR-dependent checkpoint inactivates spindle assembly by targeting CEP63. Nat Cell Biol 11: 278–285. 19182792 10.1038/ncb1835

[29] Todaro GJ, Green H (1963) Quantitative studies of the growth of mouse embryo cells in culture and their development into established lines. J Cell Biol 17: 299–313. 13985244 10.1083/jcb.17.2.299 PMC2106200

[30] Lukinavicius G, Lavogina D, Orpinell M, Umezawa K, Reymond L, et al (2013) Selective chemical crosslinking reveals a cep57–cep63–cep152 centrosomal complex. Curr Biol 23: 265–270. 23333316 10.1016/j.cub.2012.12.030

[31] Sonnen KF, Schermelleh L, Leonhardt H, Nigg EA (2012) 3D-structured illumination microscopy provides novel insight into architecture of human centrosomes. Biol Open 1: 965–976. 23213374 10.1242/bio.20122337 PMC3507176

[32] Heald R, Tournebize R, Blank T, Sandaltzopoulos R, Becker P, et al (1996) Self-organization of microtubules into bipolar spindles around artificial chromosomes in Xenopus egg extracts. Nature 382: 420–425. 8684481 10.1038/382420a0

[33] Loffler H, Fechter A, Matuszewska M, Saffrich R, Mistrik M, et al (2011) Cep63 recruits Cdk1 to the centrosome: implications for regulation of mitotic entry, centrosome amplification, and genome maintenance. Cancer Res 71: 2129–2139. 21406398 10.1158/0008-5472.CAN-10-2684

[34] Blachon S, Gopalakrishnan J, Omori Y, Polyanovsky A, Church A, et al (2008) Drosophila asterless and vertebrate Cep152 Are orthologs essential for centriole duplication. Genetics 180: 2081–2094. 18854586 10.1534/genetics.108.095141 PMC2600943

[35] Paoletti A, Moudjou M, Paintrand M, Salisbury JL, Bornens M (1996) Most of centrin in animal cells is not centrosome-associated and centrosomal centrin is confined to the distal lumen of centrioles. J Cell Sci 109 (Pt 13): 3089–3102. 10.1242/jcs.109.13.3089 9004043

[36] Tsou MF, Stearns T (2006) Mechanism limiting centrosome duplication to once per cell cycle. Nature 442: 947–951. 16862117 10.1038/nature04985

[37] Cizmecioglu O, Warnke S, Arnold M, Duensing S, Hoffmann I (2008) Plk2 regulated centriole duplication is dependent on its localization to the centrioles and a functional polo-box domain. Cell Cycle 7: 3548–3555. 19001868 10.4161/cc.7.22.7071

[38] Dodson H, Bourke E, Jeffers LJ, Vagnarelli P, Sonoda E, et al (2004) Centrosome amplification induced by DNA damage occurs during a prolonged G2 phase and involves ATM. EMBO J 23: 3864–3873. 15359281 10.1038/sj.emboj.7600393 PMC522792

[39] Loncarek J, Hergert P, Khodjakov A (2010) Centriole reduplication during prolonged interphase requires procentriole maturation governed by Plk1. Curr Biol 20: 1277–1282. 20656208 10.1016/j.cub.2010.05.050 PMC2911792

[40] Prosser SL, Samant MD, Baxter JE, Morrison CG, Fry AM (2012) Oscillation of APC/C activity during cell cycle arrest promotes centrosome amplification. J Cell Sci 125: 5353–5368. 22956538 10.1242/jcs.106096 PMC3939426

[41] Steere N, Wagner M, Beishir S, Smith E, Breslin L, et al (2011) Centrosome amplification in CHO and DT40 cells by inactivation of cyclin-dependent kinases. Cytoskeleton (Hoboken) 68: 446–458. 21766470 10.1002/cm.20523 PMC3166434

[42] Strnad P, Leidel S, Vinogradova T, Euteneuer U, Khodjakov A, et al (2007) Regulated HsSAS-6 levels ensure formation of a single procentriole per centriole during the centrosome duplication cycle. Dev Cell 13: 203–213. 17681132 10.1016/j.devcel.2007.07.004 PMC2628752

[43] Celis JE, Madsen P, Nielsen S, Celis A (1986) Nuclear patterns of cyclin (PCNA) antigen distribution subdivide S-phase in cultured cells–some applications of PCNA antibodies. Leuk Res 10: 237–249. 2419706 10.1016/0145-2126(86)90021-4

[44] Pines J, Hunter T (1991) Human cyclins A and B1 are differentially located in the cell and undergo cell cycle-dependent nuclear transport. J Cell Biol 115: 1–17. 1717476 10.1083/jcb.115.1.1 PMC2289910

[45] Lawo S, Hasegan M, Gupta GD, Pelletier L (2012) Subdiffraction imaging of centrosomes reveals higher-order organizational features of pericentriolar material. Nat Cell Biol 14: 1148–1158. 23086237 10.1038/ncb2591

