# Supplementary figures and images for: Cep63 and Cep152 Cooperate to Ensure Centriole Duplication
Source: PLoS One. 2013 Jul 30;8(7):e69986. doi: 10.1371/journal.pone.0069986 (PMC3728344; doi:10.1371/journal.pone.0069986)

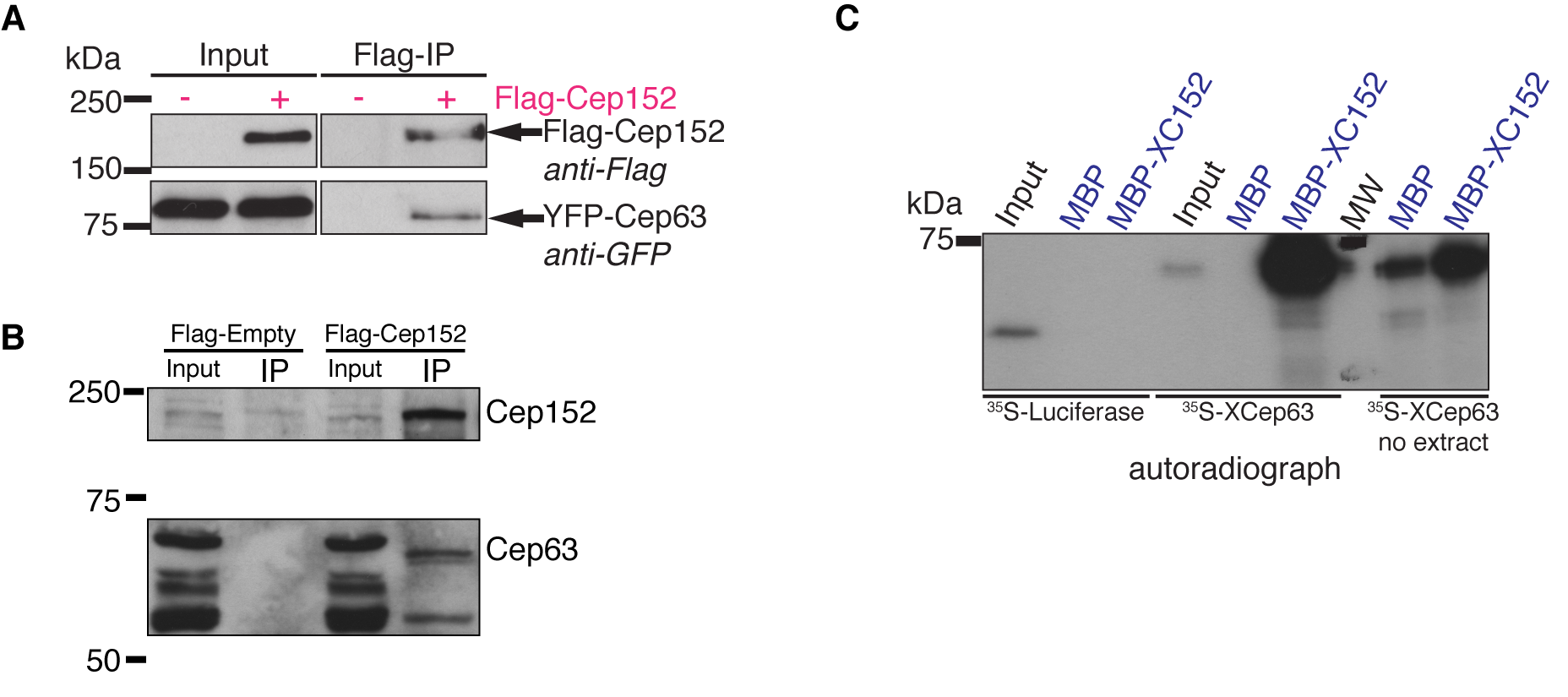

Supplement: Figure S1 — Cep63 and Cep152 interact. (A) YFP-Cep63 interacts with Flag-Cep152 in 293 HEK cells. Cells constitutively expressing YFP-Cep63 were transfected with either Flag empty vector (-) or Flag-Cep152 (+) and proteins were immuno-precipitated using anti-Flag antibody coupled beads. Input shows 10% of the whole cell lysate used for IP. Immuno-precipitated proteins were visualised by Western blotting with anti-Flag or anti-GFP antibodies. (B) Flag IP from 293 HEK cells stably expressing Flag-empty vector or Flag-Cep152 using 5 mg whole cell lysate; inputs show 100 µg whole cell lysate. Endogenous and Flag-tagged Cep152 were detected by Western blotting with anti-Cep152 antibodies (Bethyl) and endogenous Cep63 was detected using anti-Cep63 (Millipore). (C) Xenopus laevis Cep63 (XCep63) interacts with Xenopus laevis Cep152 (XCep152) in the absence of centrosomes. Amylose resin bound to MBP or MBP-XCep152 was incubated with Xenopus laevis cytostatic factor arrested egg extracts containing in vitro transcribed/translated XCep63 or Luciferase labelled with 35S methionine. Proteins were eluted by boiling in Laemmli buffer, analysed by SDS-PAGE, and detected by autoradiography. The same experiment was carried out in the absence of egg extract, using EB buffer (50 mM HEPES pH 7.5, 100 mM KCl, 2.5 mM MgCl2) as a replacement (no extract). (TIF) [file pone.0069986.s001.tif]

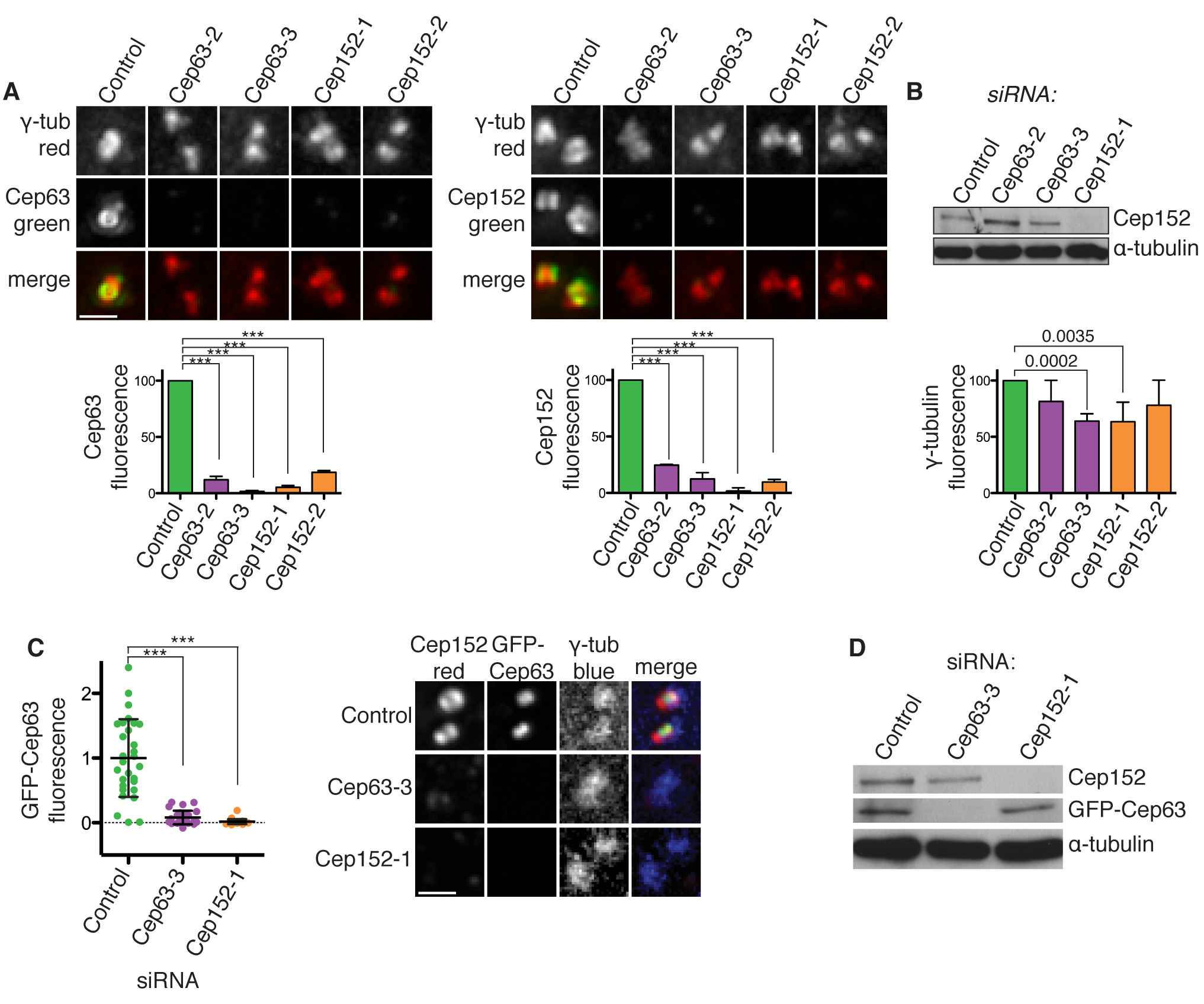

Supplement: Figure S2 — Cep63 and Cep152 are required for efficient centriole duplication and reduplication in human cells. (A) U2OS cells after Control, Cep63, or Cep152 RNAi for 96 hours, stained with anti-Centrin 2 (green) and γ-tubulin (red) antibodies and DAPI (blue). Lower panels show 3-fold enlargements of Centrin 2 staining at the centrosomes (boxed regions). Scale bar 5 µm. (B) Quantification of Centrin foci number in mitotic U2OS cells after Control, Cep63, or Cep152 RNAi from 3 independent experiments, n>20. Significant differences between the percentage of cells with fewer than 4 Centrin foci are indicated with p values calculated by a students’ t-test. (C) U2OS cells depleted of Cep63 or Cep152 by RNAi were incubated with 1.9 µg/ml aphidicolin for 72 hours. Pictures show γ-tubulin immunofluorescence (green, white in inserts) and DAPI (blue). Scale bar 5 µm. (D) Cells with more than 2 γ-tubulin foci were counted in 3 independent experiments, n = 150. (TIF) [file pone.0069986.s002.tif]

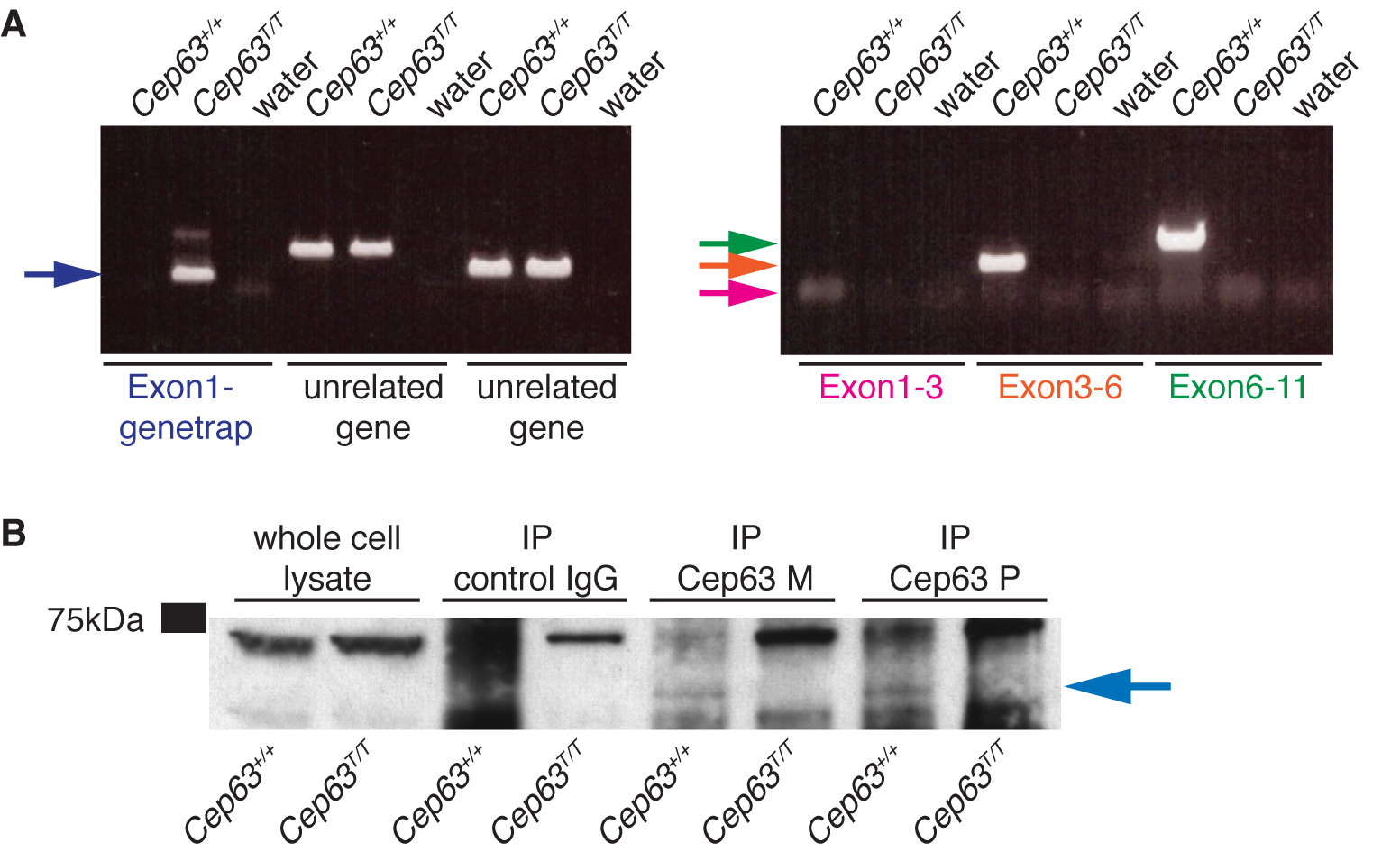

Supplement: Figure S3 — Cep63 gene-trap homozygous MEFs lack Cep63 mRNA and protein. (A) Messenger RNA from Cep63+/+ and Cep63T/T MEFs was analysed by reverse transcription-PCR using primers located within the gene-trap or within different Cep63 exons, as indicated. (B) Western blot of whole cell lysates (100 µg) and immuno-precipitates from 2 mg whole cell lysates of Cep63+/+ and Cep63T/T cell lines with pre-immune IgG (control), or two different Cep63 specific purified antibodies, M (Millipore) and P (Protein Tech Group). The Cep63 Millipore antibody was used for Western blotting. Arrow indicates a Cep63 specific band that is present in Cep63+/+, but not Cep63T/T MEFs. (TIF) [file pone.0069986.s003.tif]

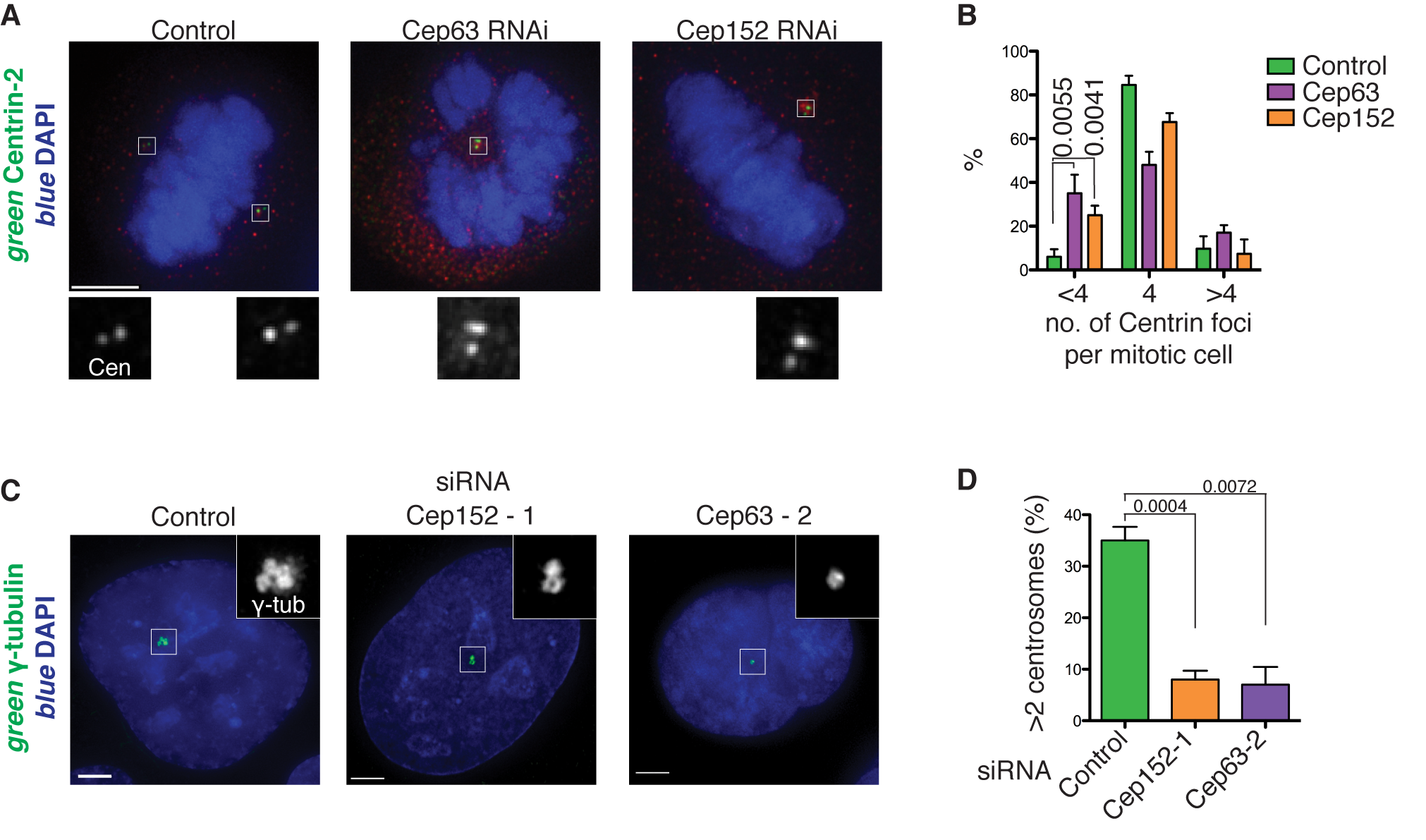

Supplement: Figure S4 — Cep63 and Cep152 are dependent on each other for centrosomal localisation. (A-B) Cep63 and Cep152 are dependent on each other for centrosomal localisation. (A) Control, Cep63 (63-2 and 63-3), or Cep152 (152-1 and 152-2) RNAi was carried out over 4 days in U2OS cells and the fluorescence intensities of Cep63 (left), Cep152 (middle), and γ-tubulin (right) were measured at the centrosome in multiple cells (n>25) in 3 experiments. The graphs show the mean fluorescence intensities normalised to the mean of the control population and the standard deviation, and p values are indicated above (*** denotes p<0.0001). Images of centrosomes from these cells are shown, from cells stained with anti-Cep63 (left) or Cep152 (right) in green and γ-tubulin (red). (B) Cep63 RNAi does not affect total levels of Cep152 protein. Western blot of whole cell lysates of U2OS after 4 days of RNAi treatment with the siRNAs indicated, showing endogenous Cep152 and α-tubulin as a loading control. (C) Quantification of GFP-Cep63 fluorescence intensity (GFP direct fluorescence) at the centrosomes of U2OS cells expressing GFP-Cep63 and transfected with Control, Cep63 (63-3), or Cep152 (152-1) siRNAs, n = 30. *** Indicates a p value of <0.0001 calculated using a students’ t-test. Representative pictures of centrosomes from these cells stained with anti-Cep152 (red) and γ-tubulin (blue) antibodies are shown in the right hand panel. GFP fluorescence is shown in green. Scale bar 1 µm. (D) Cep152 RNAi does not affect total levels of GFP-Cep63 protein. Western blot of whole cell lysates from cells used in (C) using anti-Cep152, GFP, and α-tubulin antibodies. (TIF) [file pone.0069986.s004.tif]

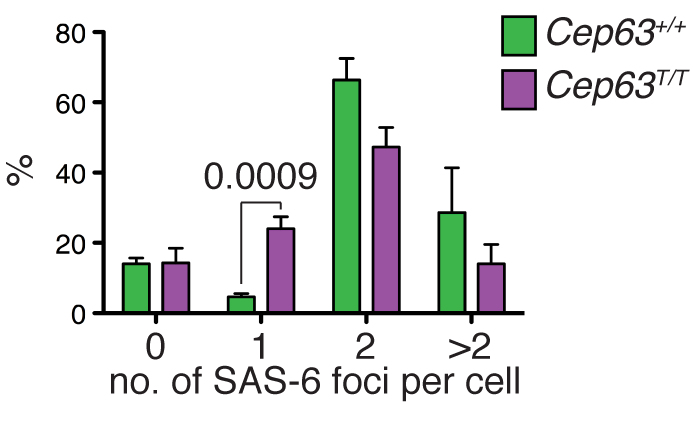

Supplement: Figure S5 — Impaired SAS-6 recruitment in Cep63 deficient mouse cell lines. Cep63+/+ or Cep63T/T MEF cell lines, immortalised by SV40 large T antigen expression, were incubated with aphidicolin (2 µg/ml) for 24 hours, then fixed and stained with anti-HsSAS-6 antibodies. The number of SAS-6 foci per cell was counted for three Cep63+/+ cell lines and three Cep63T/T littermate controls, n >100. The difference in percentage of cells with only one SAS-6 focus was significant (p = 0.0009, students’ t-test). (TIF) [file pone.0069986.s005.tif]

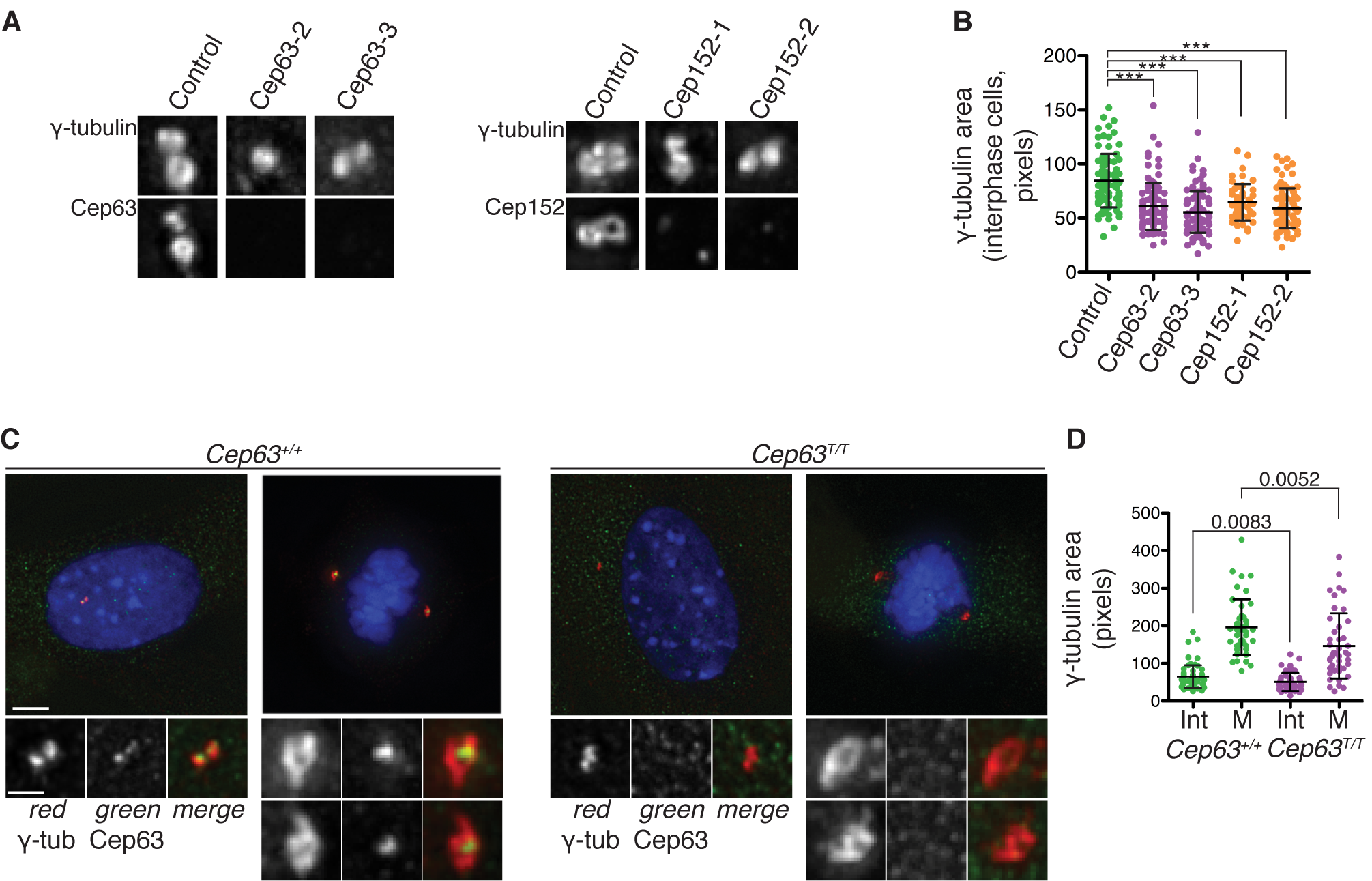

Supplement: Figure S6 — Reduced centrosomal γ-tubulin after Cep63–Cep152 depletion. (A-B) Depletion of Cep63 and Cep152 leads to a slight reduction in PCM size. (A) Images of centrosomes from U2OS cells treated with control, or two different Cep63 (63-2, 63-3) or Cep152 (152-1, 152-2) siRNAs, stained with anti-γ-tubulin and Cep63 or Cep152 antibodies. Scale bar 5 µm. (B) γ-tubulin area was measured in interphase cells, from z-stack projections, using Image J software in number of pixels, n >25. (C-D) Cep63T/T primary MEFs have a slight reduction in PCM size in both interphase and mitosis. (C) Images of interphase and mitotic Cep63+/+ or Cep63T/T primary MEFs at passage 4, stained with anti- γ-tubulin (red) and Cep63 (green) antibodies and DAPI (blue). Scale bars are 5 µm for large panels and 1 µm for small panels, which show enlargements of each centrosome. (D) γ-tubulin area was measured in interphase and mitotic cells, from z-stack projections, using Image J software in number of pixels, n >50 for interphase cells and n >43 for mitotic cells. (TIF) [file pone.0069986.s006.tif]
