# Supplementary material for: Cep63 and Cep152 Cooperate to Ensure Centriole Duplication
Source: PLoS One. 2013 Jul 30;8(7):e69986. doi: 10.1371/journal.pone.0069986 (PMC3728344; doi:10.1371/journal.pone.0069986)
Supplement: Supporting Information S1 — (PDF) [file pone.0069986.s007.pdf]

Supporting Information

Cep63 and Cep152 cooperate to ensure centriole duplication.

Brown *et al.* 2013

Supplementary Materials and Methods

Supplementary plasmids

Xenopus laevis Cep152 was subcloned from IMAGE: 5084879 into the pMALc4x bacterial expression vector (NEB). *Xenopus laevis* Cep63 was expressed from cDNA (BC088974 *Xenopus laevis* hypothetical LOC496369) in a pCS2 expression vector.

Xenopus laevis egg extracts and pull down assays

Cytostatic factor (CSF) arrested extracts from *Xenopus laevis* eggs, arrested in meiosis II, were made as described previously [1]. SP6 quick-coupled transcription-translation reticulocyte lysate (TNT, Promega) was used to produce *Xenopus laevis* Cep63 protein (XCep63) in the presence of ^{35}S-methionine (Promix, Amersham). MBP and MBP-*Xenopus laevis* Cep152 (XCep152) were expressed in in BL21 CodonPlus (DE3) RIL *E. coli* (Stratagene) at 16 ˚C for 3 hours after induction with 0.3 mM IPTG (Isopropyl β-D-1-thiogalactopyranoside), and purified using amylose resin according the manufacturer's protocol (NEB). For pull down assays, 2 µg MBP or MBP-XCep152 was coupled to 25 µl amylose resin per reaction, then incubated with 15 µl TNT transcription translation mix containing ^{35}S-XCep63 or ^{35}S-Luciferase +/- 150 µl CSF extract (as indicated), diluted to a total volume of 0.2 ml with EB (50 mM HEPES pH7.5, 100 mM KCl, 2.5 mM $MgCl_2$), and incubated for 1 hour at 4 ˚C. Resin was washed extensively with PBS 0.25% Igepal and PBS, then proteins were eluted by boiling in Laemmli buffer, then analysed by SDS-PAGE and autoradiography.

Cep63 immunoprecipitation

For IP, 10 µg of pre-immune purified rabbit IgG (control), anti-Cep63 (Millipore), or anti-Cep63 (ProteinTech Group) was added to 2 mg cell lysate (lysis buffer: 50 mM Tris HCl, pH 7.4, 150 mM NaCl, 1 mMEDTA, 1% Triton X100) plus Complete protease inhibitors (Roche) and incubated for 2 hours at 4 ˚C. 60 µl protein A/G beads (Stratagene) were added to each sample and incubated for 1 hour at 4 ˚C, then washed in lysis buffer followed by PBS. Proteins were eluted using Laemmli buffer without β-mercaptoethanol, at room temperature, then analysed by SDS-PAGE and Western blotting

with anti-Cep63 (Millipore) and an anti-rabbit light chain specific secondary antibody coupled to HRP (Cell Signaling).

Supplementary References

1. Desai A, Murray A, Mitchison TJ, Walczak CE (1999) The use of Xenopus egg extracts to study mitotic spindle assembly and function in vitro. Methods Cell Biol 61: 385-412.
